# IRG1–itaconate axis in immunometabolism: mechanistic roles and therapeutic potential in inflammatory diseases

**DOI:** 10.3389/fimmu.2026.1767601

**Published:** 2026-02-10

**Authors:** Yang Liu, Pengyu Zhang, Weijie Kong, Jinghui Li, Haoqiang Yang, Hengqi Bai, Gexu Fan, Xinfang Cao, Yanjun Li

**Affiliations:** 1 Third Hospital of Shanxi Medical University, Shanxi Bethune Hospital, Shanxi Academy of Medical Sciences, Tongji Shanxi Hospital, Taiyuan, China; 2 Department of Hepatobiliary Surgery, Shanxi Bethune Hospital, Shanxi Academy of Medical Sciences, TongjiShanxi Hospital, Third Hospital of Shanxi Medical University, Taiyuan, Shanxi, China

**Keywords:** ACOD1/itaconate axis, immunometabolism, inflammation, Keap1-Nrf2, macrophage activation, SDH, TET2, therapeutic targeting

## Abstract

Cells can produce various metabolites, and both immune cells and the immune microenvironment are profoundly influenced by these metabolites. By reshaping the metabolic state of immune cells via metabolites, the host immune response can be effectively regulated, further impacting their behavior in inflammation. Itaconate, as a bypass metabolite of the tricarboxylic acid (TCA) cycle, has long been regarded as a small molecule involved in energy metabolism. However, recent studies reveal its production depends on immune response gene 1 (IRG1), which encodes aconitate decarboxylase. Under the stimulation of inflammation, the expression of IRG1 is significantly upregulated, leading to the rapid accumulation of itaconate within immune cells (especially macrophages), thus making it a key link between metabolism and immune response. Evidence indicates that macrophages are the cell type extensively synthesizing itaconate during M1 polarization driven by potent inflammatory signals (e.g., LPS stimulation). Concurrently, itaconate participates in regulating immune tolerance to cancer therapy via the transmembrane transporter SLC13A3. Under different pathological contexts the IRG1- itaconate axis exhibits distinct dynamic regulatory characteristics: During acute inflammation, itaconate limits excessive release of pro-inflammatory factors and reduces tissue damage by inhibiting succinate dehydrogenase (SDH), activating the Keap1-Nrf2 antioxidant pathway, blocking the ATF3/IκBζ-dependent pro-inflammatory program, and regulating the TET2-mediated epigenetic network. In chronic inflammation or certain tumor microenvironments, however, it may indirectly promote immunosuppression by inhibiting antigen presentation and weakening T cell cytotoxic effects. This bidirectional and environment-dependent nature makes it a key entry point for understanding the maintenance of immune homeostasis, inflammatory regulation and disease progression. This review systematically examines the production mechanisms, biochemical properties, central signaling pathways, and cross-cell effects of Itaconate as an immunomodulatory metabolite. It emphasizes its dual role in regulating inflammatory responses through multiple signaling axes and its contrasting behaviors in different disease contexts. By elucidating its molecular mechanisms, this study aims to provide novel theoretical foundations and potential therapeutic strategies for precision interventions in inflammatory diseases, while outlining future research directions and the clinical translation potential of itaconate-based approaches.

## 1 Introduction

**Figure 1:**
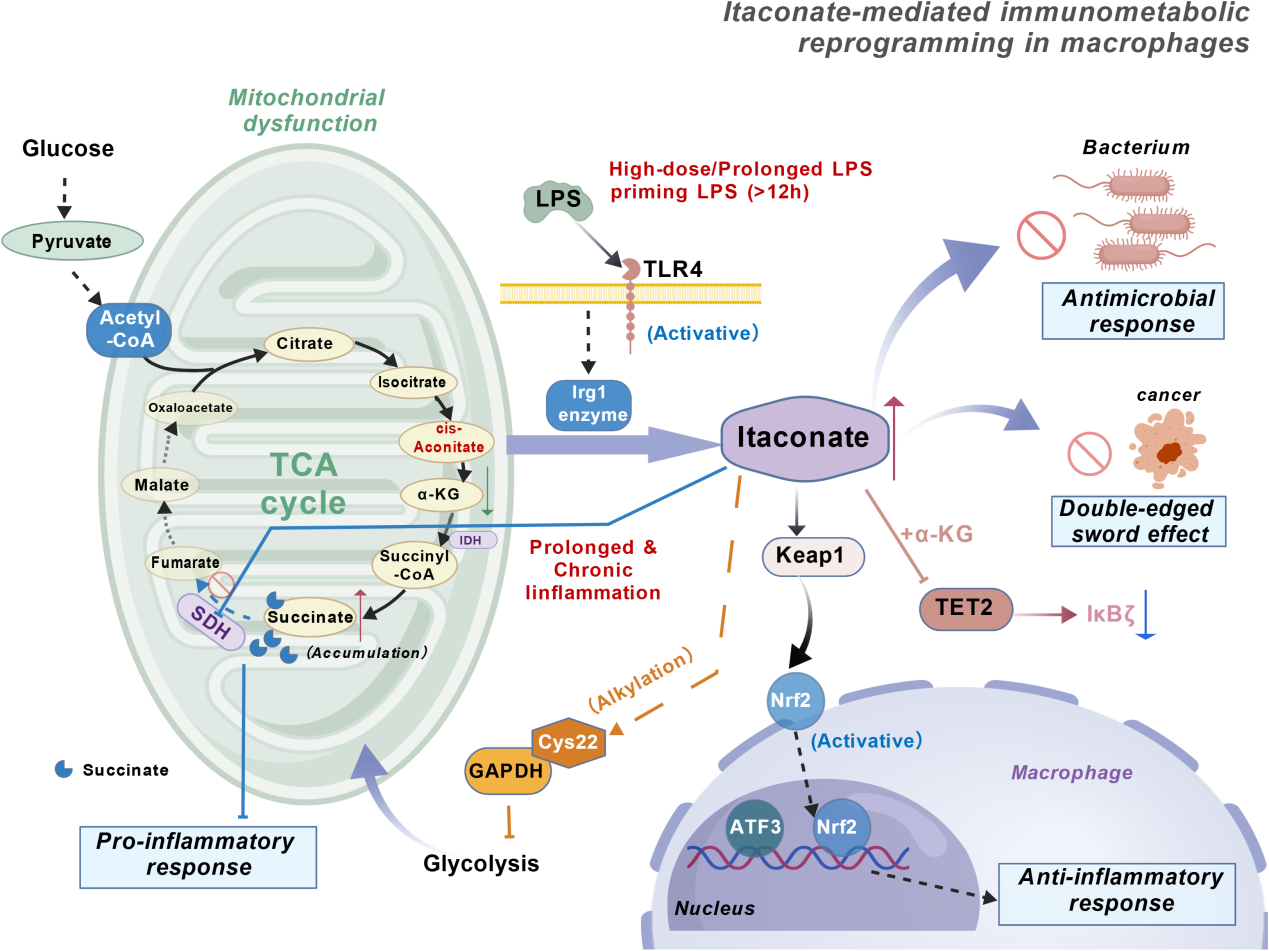
Itaconate-dependent immunometabolic reprogramming in macrophages. Upon high-dose and/or prolonged LPS priming (>12 h) through TLR4, macrophages induce the Irg1 enzyme, which converts cis-aconitate (from the mitochondrial TCA cycle) into itaconate. In mitochondria, itaconate inhibits SDH, leading to succinate accumulation and associated pro-inflammatory responses under conditions of mitochondrial dysfunction. In parallel, itaconate exerts anti-inflammatory programs by modifying Keap1 to activate Nrf2, promoting nuclear transcriptional responses (including ATF3) that drive an anti-inflammatory response. Itaconate also alkylates GAPDH at Cys22, thereby inhibiting GAPDH activity and reshaping inflammatory metabolism, and can modulate α-KG–dependent TET2 signaling, resulting in decreased IκBζ expression. Collectively, these pathways contribute to antimicrobial effects and a context-dependent “double-edged sword” outcome during prolonged or chronic inflammation. Created with BioGDP.com. TLR4, Toll-like receptor 4; TCA, tricarboxylic acid; LPS, lipopolysaccharide; Irg1, immune-responsive gene 1; SDH, succinate dehydrogenase; α-KG, alpha-ketoglutarate; Keap1, Kelch-like ECH-associated protein 1; Nrf2, nuclear factor erythroid 2–related factor 2; ATF3, activating transcription factor 3; GAPDH, glyceraldehyde-3-phosphate dehydrogenase; TET2, ten-eleven translocation 2; IκBζ, inhibitor of NF-κB zeta; Acetyl-CoA, acetyl coenzyme A; Cys22, cysteine residue at position 22 of GAPDH.

itaconate is a metabolic product derived from the tricarboxylic acid (TCA) cycle, catalyzed by the metabolic enzyme aconitate decarboxylase 1 (ACOD1), encoded by the immune response gene 1 (IRG1) (1, 2)(**Figure 1**). The TCA cycle serves as the primary metabolic pathway for energy production in eukaryotes, and its metabolites exert significant influence on immune cells and the immune microenvironment (3). Macrophages constitute a major component of the innate immune barrier; under physiological conditions, they can be activated by various infections and injuries, undergoing metabolic reprogramming to exert effector functions (4, 5). Itaconate and its the biosynthetic enzyme IRG1 are highly conserved throughout evolution, retaining their immunometabolic regulatory functions from invertebrates to mammals, forming an ancient yet pivotal component of the innate immune system (1). IRG1 encodes aconitate decarboxylase (ACOD1), which catalyzes the decarboxylation of cis-aconitate to produce Itaconate (2, 4, 6). Itaconate is highly expressed in activated macrophages and can influence changes in macrophage metabolites and mitochondrial respiration (7). In 2011, Shin et al. observed significantly elevated Itaconate levels in lung tissues of mice infected with Mycobacterium tuberculosis, suggesting that the pathogen may induce endogenous production (8). Subsequently, Strelko et al. demonstrated that LPS or IFN-γ stimulation rapidly induces massive synthesis and release of Itaconate by activated macrophages (9). In 2013, Micheluzzi et al. elucidated the biosynthetic pathway of Itaconate in mammals (2). Since then, Itaconate has been regarded as a central mediator linking immunity, metabolism, and inflammation, with its derivatives gaining increasing attention for their potential in treating inflammatory and immune-related diseases (4, 9, 10). Research on Itaconate and its derivatives holds significant implications for the treatment of inflammatory and immune-related disorders. In recent years, the combined effects of multiple risk factors and lifestyle changes have gradually increased the incidence of inflammatory diseases, significantly impacting patients’ long-term quality of life (5). Itaconate exerts anti-inflammatory and antioxidant effects through diverse mechanisms, playing a pivotal role in immune-related disorders, infectious diseases, and certain malignancies (11). With the establishment of its dual “immune-metabolic” identity (12), its antibacterial and immunomodulatory potential has rapidly gained prominence (13). This review systematically summarizes the aforementioned properties of Itaconate and further explores its translational prospects in inflammatory therapies, providing a theoretical foundation for subsequent research and clinical applications (14).

## 2 The structure of itaconate

**Figure 2:**
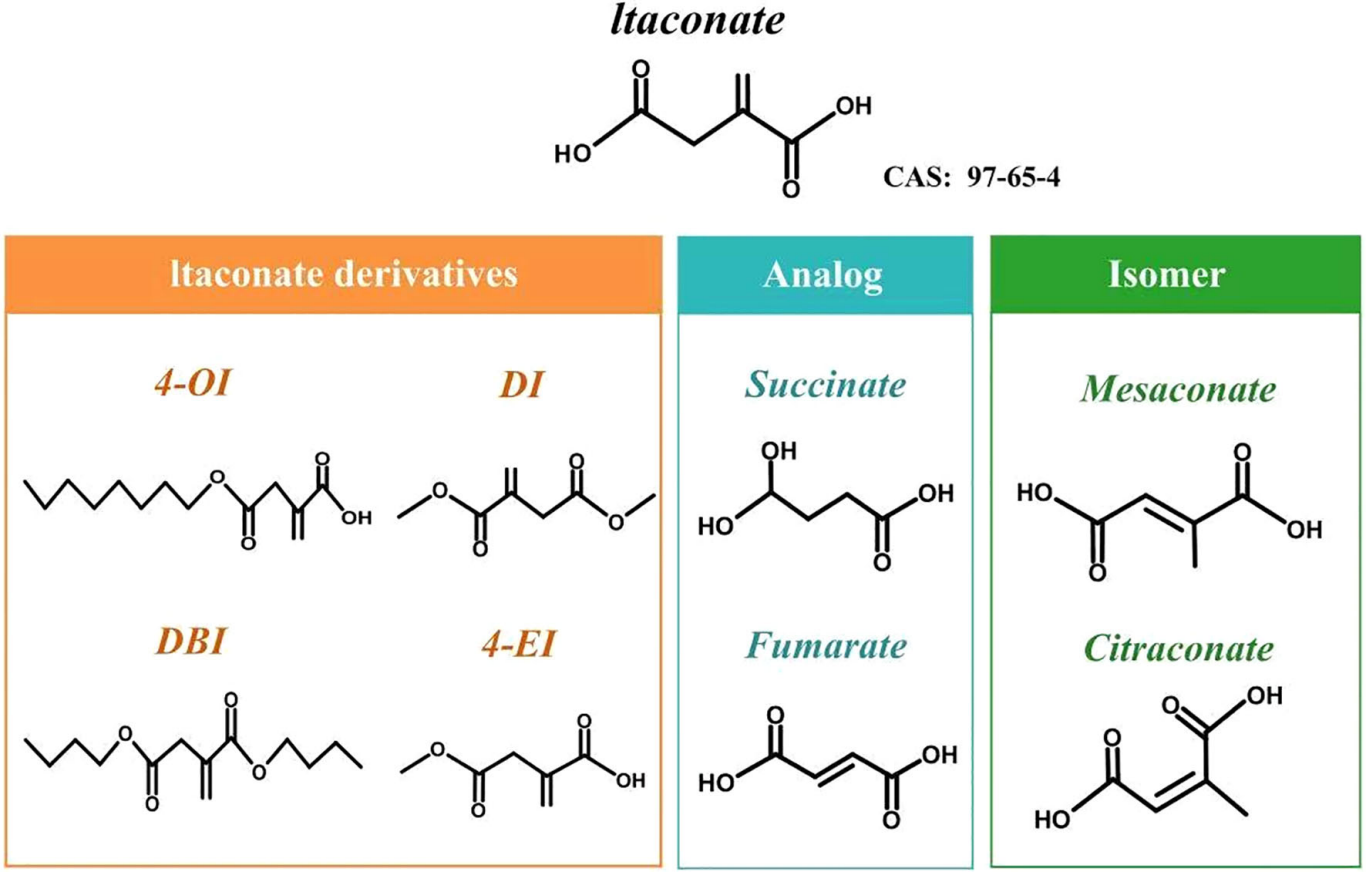
Chemical structures of itaconate, its derivatives, selected analogs, and geometric isomers.The figure illustrates the parent metabolite itaconate (CAS: 97-65-4), commonly used cell-permeable itaconate derivatives (4-OI, DI, DBI, and 4-EI), representative structural analogs in the tricarboxylic acid cycle (succinate and fumarate), and related isomers (mesaconate and citraconate) for comparison. 4-OI, 4-octyl itaconate; DI, dimethyl itaconate; DBI, dibutyl itaconate; 4-EI, 4-ethyl itaconate.

The molecular formula of itaconate is C_5_H_6_O_4_, and it has a density of 1.57 g cm^-^³. It is highly soluble in water (≥200 g L^-^¹ at 20 °C), ethanol, and acetone, slightly soluble in benzene and chloroform, and appears as a white crystalline powder (15). It remains stable when stored at room temperature. Structurally, it is a five-carbon dicarboxylic acid with an α, β-unsaturated alkene (3), sharing the 2-methylenebutanedioic acid skeleton with one conjugated double bond and two carboxyl groups. Its structure and chemistry resemble, in many ways, other metabolites such as phosphoenolpyruvate, succinate, malonic acid, and fumarate. These similarities offer useful reference points for studying its antibacterial and immunomodulatory effects (7). The unsaturated double bond and two active carboxyl groups in itaconate enable it to undergo various chemical reactions (16), with esterification being particularly significant (17). The itaconate molecule contains two carboxyl groups, which can undergo monoesterification or diesterification. This results in commonly used ester derivatives such as 4-octyl itaconate (4-OI), dimethyl itaconate (DI), monomethyl itaconate (MMI), and dibutyl itaconate (DBI) (**Figure 2**). Among these, 4-octyl itaconate and dimethyl itaconate are frequently used as substitutes for itaconate due to the high membrane permeability and electrophilicity of their derivatives. They serve as Nrf2 activators, inhibiting pro-inflammatory factors like IL-1β and IL-12p40 to mimic itaconate’s biological effects *in vitro* and *in vivo (*11, 18). However, the electrophilicity, permeability, or other chemical differences of itaconate derivatives may induce effects not observed with endogenous itaconate (19). The utilization of itaconate derivatives represents an important area for further investigation, potentially leading to novel itaconate-based therapeutics.

## 3 Immunomodulatory properties of itaconate

**Figure 3:**
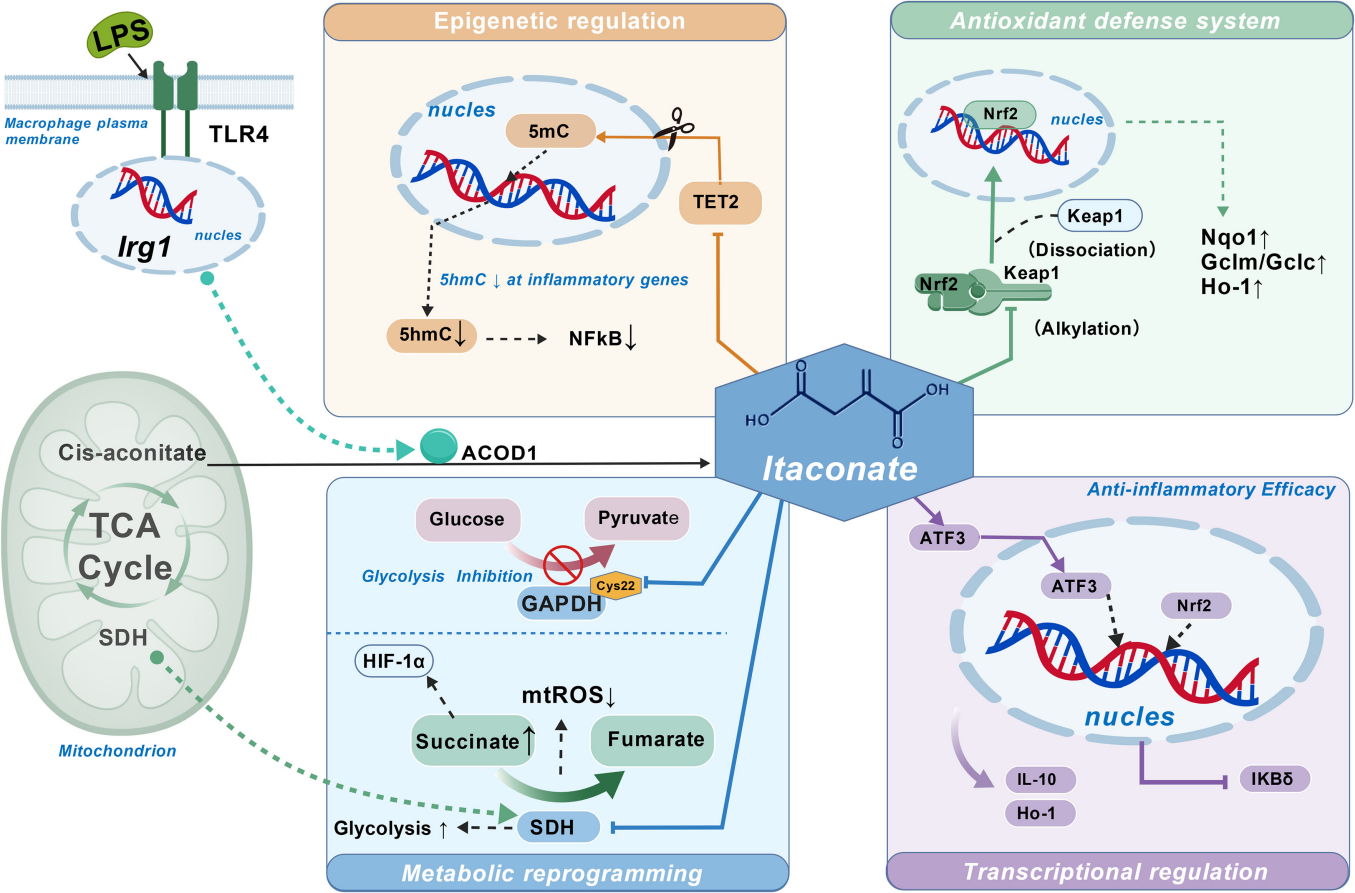
The role of itaconate in immunoregulatory properties. In macrophages, LPS stimulation through TLR4 induces Irg1, and ACOD1 converts mitochondrial cis-aconitate in the TCA cycle to itaconate. Itaconate coordinates immunoregulation through four interconnected modules. (i) Epigenetic regulation: itaconate modulates TET2 activity and the balance between 5mC and 5hmC, leading to decreased 5hmC at inflammatory genes and reduced NFκB signaling. (ii) Antioxidant defense system: itaconate alkylates Keap1, promoting Keap1 dissociation and Nrf2 nuclear translocation, thereby upregulating antioxidant/cytoprotective genes (Nqo1↑, Gclm/Gclc↑, Ho-1↑). (iii) Metabolic reprogramming: itaconate inhibits SDH, resulting in succinate↑ and altered conversion to fumarate, reduced mtROS↓, and modulation of HIF-1α; it also inhibits glycolysis by alkylating GAPDH (Cys22), affecting the glucose–pyruvate axis. (iv) Transcriptional regulation (anti-inflammatory efficacy): itaconate promotes ATF3-associated transcriptional programs (with Nrf2), enhances anti-inflammatory mediators (IL-10, Ho-1), and suppresses IKBδ, collectively contributing to anti-inflammatory outcomes. Created with BioGDP.com. LPS, lipopolysaccharide; TLR4, Toll-like receptor 4; Irg1, immune-responsive gene 1; ACOD1, aconitate decarboxylase 1; TCA, tricarboxylic acid; SDH, succinate dehydrogenase; mtROS, mitochondrial reactive oxygen species; HIF-1α, hypoxia-inducible factor 1α; GAPDH, glyceraldehyde-3-phosphate dehydrogenase; Cys22, cysteine residue at position 22 of GAPDH; TET2, ten-eleven translocation 2; 5mC, 5-methylcytosine; 5hmC, 5-hydroxymethylcytosine; NFκB, nuclear factor κB; Keap1, Kelch-like ECH-associated protein 1; Nrf2, nuclear factor erythroid 2–related factor 2; Nqo1, NAD(P)H quinone dehydrogenase 1; Gclm/Gclc, glutamate–cysteine ligase modifier/catalytic subunits; Ho-1, heme oxygenase-1; ATF3, activating transcription factor 3; IL-10, interleukin-10; IKBδ, inhibitor of NF-κB delta.

Extensive research indicates that under microbial stimulation from viruses, bacteria, or fungi, itaconate significantly accumulates in macrophages, dendritic cells, and neutrophil subsets, while the expression of its rate-limiting enzyme IRG1 is highly induced. As a key metabolite in immune metabolic reprogramming, itaconate has become a focal point of research in this field due to its multifaceted immunomodulatory functions. However, its full biological effects and molecular mechanisms remain to be systematically explored. This review provides a comprehensive examination of itaconate’s immune functions, integrating recent advances to focus on its metabolic regulatory roles as an SDH inhibitor, TET2 inhibitor, Nrf2 activator, and glycolysis modulator. It examines its regulatory mechanisms within the ATF3/IκBζ transcription axis, explores its action patterns across various signaling networks, and explores its potential for translational applications in immune-related diseases (**Figure 3**).

### 3.1 Itaconate: an endogenous inhibitor of SDH

SDH (succinate dehydrogenase, also known as mitochondrial respiratory chain complex II) is located in the inner mitochondrial membrane and serves as a key bifunctional enzyme linking the tricarboxylic acid cycle to the electron transport chain. In LPS-activated macrophages, it catalyzes the oxidation of succinate to fumarate and generates FADH_2_, directly transferring electrons to ubiquinone (20). This process simultaneously sustains the TCA cycle and participates in oxidative phosphorylation (21). Recent studies indicate that changes in SDH activity during inflammation are closely linked (4, 7). As early as 2016, researchers demonstrated through *in vivo* and *in vitro* experiments that itaconate suppresses inflammation by inhibiting SDH-mediated succinate oxidation (4, 20). This mechanism stems from itaconate’s structural similarity to succinate, enabling competitive inhibition of the SDH enzyme’s active site. This reduces succinate oxidation to fumarate, thereby preventing the production of mitochondrial reactive oxygen species (mtROS) driven by Complex I. mtROS inhibits prolyl hydroxylase (PHD), thereby promoting HIF-1α and IL-1β production (22–24). As a result, suppressing mtROS accumulation restores PHD activity and lowers HIF-1α and IL-1β levels. This, in turn, attenuates the transcriptional expression of glycolytic rate-limiting enzymes and proinflammatory genes (24). These studies reveal SDH’s role as a key node in inflammatory metabolic regulation and provide crucial theoretical support for understanding the anti-inflammatory mechanism of itaconate.

Extensive research has shown that cell-permeable itaconate derivatives significantly suppress expression of multiple pro-inflammatory factors, including IL-1β, IL-6, IL-12, and IL-18 (11, 20, 24). In activated macrophages, IRG1 induction elevates itaconate levels and promotes succinate accumulation, highlighting the critical role of itaconate in macrophage effector functions (23). Further experimental studies revealed that under LPS stimulation, no succinate accumulation was observed in Irg1^-^/^-^ mouse bone marrow-derived macrophages (BMDMs), suggesting preserved SDH activity (20). Concurrently, Irg1 deficiency enhanced nitric oxide production and promoted the release of proinflammatory cytokines (including IL-6, IL-1β, IL-18, and IL-12p70) compared to wild-type cells, further supporting the notion that itaconate exerts anti-inflammatory effects by inhibiting SDH (23). Notably, itaconate induces innate immune tolerance, a state of immunoparalysis resulting from excessive SDH inhibition. This effect can be reversed by β-glucans derived from fungal and certain Gram-positive bacterial cell walls, which downregulate IRG1 transcription and thereby reduce competitive inhibition of SDH (13, 18). These findings suggest that the IRG1-Itaconate-SDH axis not only contributes to inflammation suppression but also forms an immunometabolic circuit with both negative and positive feedback regulation, playing a crucial role in maintaining the dynamic equilibrium of inflammatory responses.

In summary, itaconate accumulation promotes succinate buildup by inhibiting SDH activity, which is one of its most critical metabolic features (20, 23). Beyond regulating host metabolism, itaconate-mediated SDH inhibition also impacts gut microbiota ecology. Elevated succinate levels not only suppress the growth of short-chain fatty acid (SCFA)-producing bacteria such as Clostridium butyricum (24–26) but also indirectly weaken their role in maintaining intestinal barrier function. Enriched succinate also provides an additional carbon source for opportunistic pathogens (e.g., certain Enterobacteriaceae), further exacerbating ecological imbalance (27–29). Collectively, the itaconate-SDH inhibition axis not only regulates host immune cells but also profoundly impacts gut symbionts, methanogenic archaea, and alveolar colonizing bacteria (30). Itaconate inhibits SDH, leading to succinate buildup, which affects macrophage metabolism and immune responses. This mechanism helps suppress inflammation and may offer a therapeutic strategy, particularly for immune-related diseases, by controlling excessive inflammation and restoring immune balance.

### 3.2 Itaconate activates the Keap1–Nrf2 signaling pathway

The Keap1-Nrf2-ARE pathway plays a central role in maintaining cellular redox homeostasis. Its downstream gene products participate in antioxidant, anti-inflammatory, and metabolic reprogramming through mechanisms such as scavenging reactive oxygen species (ROS), inhibiting NLRP3 inflammasome and NF-κB signaling, and detoxifying electrophilic metabolites (31–35). Among these, Nrf2 is the key transcription factor in this pathway, regulating antioxidant gene expression by binding to the ARE element within the promoter regions of target genes (36–38). Under physiological conditions, Nrf2 is regulated by the KEAP1 protein, which binds to it in the cytoplasm and mediates its degradation (39–41). When cells undergo oxidative stress, the interaction between Keap1 and Nrf2 becomes unstable, resulting in the release of Nrf2 (39–41). Nrf2 translocates to the nucleus where it forms heterodimers with sMaf. The Nrf2-Maf complex suppresses pro-inflammatory genes by directly binding to IL-1β and IL-6 promoters and inhibiting the recruitment of RNA polymerase II (42, 43). It also activates downstream genes including NQO1 (NAD(P)H quinone dehydrogenase 1), GCLC/GCLM (glutamate–cysteine ligase modifier/catalytic subunits), and HO-1 (heme oxygenase-1), which are involved in antioxidant and anti-inflammatory processes (40, 41). This facilitates the clearance of reactive oxygen species (ROS) and reactive nitrogen species (RNS), thereby reducing oxidative stress levels and indirectly suppressing inflammatory responses (44, 45).

Evidence indicates that Irg1 deficiency significantly impairs Nrf2 activity in LPS-activated macrophages (46–48). This finding highlights itaconate’s crucial role in Nrf2 regulation and its significant contribution to suppressing oxidative stress and inflammation, potentially revealing further critical functions within macrophages (49). As an electrophilic small molecule (50), itaconate can alkylate cysteine residues (Cys151, Cys257, Cys288) on the KEAP1 protein (11, 49). This alters the spatial structure of Keap1, releasing its binding to Nrf2. Consequently, Keap1 can no longer mark Nrf2 as a “protein destined for degradation, “ leading to Nrf2 accumulation within the cell and its subsequent translocation into the nucleus. This process increases the expression of downstream genes involved in antioxidant and anti-inflammatory processes. Recent studies have revealed that Nrf2 activation is a key step in the inhibition of IL-1β expression by 4-Octyl Itaconate (4-OI), a process dependent on the reactivity of Keap1-Cys residues and the presence of Nrf2 (51). A 2024 study showed that itaconate inhibits Nrf2 activation by stabilizing KEAP1, which induces ferroptosis in triple-negative breast cancer stem cells and suppressing their proliferation. This suggests its potential use in cancer stem cell therapy (52, 53). Concurrently, a 2023 study reported that ursodeoxycholic acid alleviates oxidative stress damage and improves cardiac function by activating the Nrf2/HO-1 pathway in an experimental autoimmune myocarditis model, suggesting potential cardioprotective effects of targeting the Nrf2 axis (54). Similarly, itaconate and its derivatives exert antioxidant and anti-inflammatory effects by modifying Keap1 and activating Nrf2 signaling (49, 55). However, further investigation is needed to determine their role in myocarditis. Collectively, these studies support itaconate’s multi-level regulatory role in the Nrf2 signaling pathway.

Recent studies indicate that prolonged or excessive Nrf2 activation may lead to adverse biological effects, such as skin hyperkeratosis, epithelial thickening, and apoptosis under certain conditions (56, 57). This suggests that Nrf2 activation should be maintained with moderation and caution. Overall, itaconate exerts multi-layered and context-dependent biological effects by regulating Nrf2 and its downstream gene networks. Crucially, this action is not unidirectional inhibition but rather resembles an “inflammation sensing” process exhibiting dynamic regulation across different immune states. Thus, the IRG1/itaconate pathway serves not only as a key component of inflammatory negative regulation but also as an immunometabolic regulatory axis with bidirectional modulation potential. Itaconate regulates the Nrf2 pathway by modifying the KEAP1 protein, leading to the activation of antioxidant and anti-inflammatory genes. This helps control oxidative stress and inflammation. However, excessive Nrf2 activation can have negative effects, so its modulation needs to be balanced. Itaconate’s ability to both suppress inflammation and regulate immune responses makes it a promising therapeutic option for conditions like cancer and autoimmune diseases.

### 3.3 Itaconate-mediated modulation of the ATF3/IκBζ pathway

ATF3 (cAMP response element-binding protein 3) is a stress-response transcription factor typically induced when cells undergo oxidative stress or inflammatory stimulation (58). Research shows that ATF3 suppresses IκBζ expression (nuclear factor κB inhibitor zeta), a key factor in the NF-κB signaling pathway that promotes the production of pro-inflammatory cytokines such as IL-6. As a result, ATF3 indirectly inhibits NF-κB pathway activation by inhibiting IκBζ expression, thereby attenuating inflammatory responses (59). Itaconate, a naturally occurring compound with electrophilic properties, induces electrophilic stress by reacting with intracellular sulfhydryl groups, thereby activating ATF3 expression. Itaconate upregulates ATF3 expression through this mechanism, as studies have shown, thereby suppressing IκBζ expression and attenuating NF-κB-mediated inflammation. This suggests it may help maintain immune homeostasis by regulating the ATF3/IκBζ axis. *In vivo* studies confirm that itaconate alleviates IL-17-IκBζ-driven skin inflammatory pathology in mouse models by enhancing ATF3 expression and suppressing IκBζ, demonstrating its potential for treating autoimmune skin diseases (60). Recent studies show that itaconate improves intestinal immune balance, enhances the intestinal barrier function, and promotes gut homeostasis via the ATF3/IκBζ axis (61–64). Itaconate regulates inflammation by activating ATF3, which suppresses IκBζ and modulates the NF-κB pathway, maintaining immune balance. Its ability to reduce inflammation in autoimmune diseases and support intestinal health makes it a promising therapeutic for autoimmune disorders.

### 3.4 Itaconate inhibits TET2 activity

TET2 (Ten-Eleven Translocation 2) belongs to the α-ketoglutarate (α-KG)-dependent dioxygenase family, alongside TET1 and TET3, collectively known as the TET family (65). TET2 remodels the epigenetic landscape of inflammation-related enhancers and promoters by oxidizing 5-mC to 5-hmC/5-fC/5-caC, thereby restricting pro-inflammatory transcription and promoting inflammation resolution (65–67). Under inflammatory conditions, the IRG1-fumaric acid axis is activated. which it competitively binds to α-KG and inhibits TET2 activity. This downregulates several TET2-dependent inflammatory genes, reduces the abundance of 5-hmC at key gene sites, including IκBζ, and restricts NF-κB/STAT-mediated proinflammatory programs at the epigenetic level, thereby mitigating acute inflammatory responses and reducing endotoxin-induced mortality (68–70). At the same time, itaconate releases Nrf2 by alkylating Keap1 and induces ATF3 to inhibit IκBζ, forming a multi-tiered regulatory network that includes Nrf2 antioxidant signaling, TET2 epigenetic braking, and ATF3/IκBζ transcriptional capping. This synergistically prevents over-inflammation (70, 71).

In LPS-induced sepsis models, TET2^-^/^-^ mice did not show the anti-inflammatory effects of itaconate, indicating TET2’s critical role in itaconate-mediated immune regulation (72). Recent studies reveal that TET2-mutant hematopoietic cells in the tumor microenvironment reprogram macrophages to an ‘antigen-presenting’ phenotype. These reprogrammed macrophages activate CD8^+^ T cells more effectively under IFN-γ/immunotherapy (ICT) settings, thereby enhancing immunotherapy response and prognosis. This offers new insights into the relationship between chronic inflammation, tumor progression, and immunotherapy response (73–75). Furthermore, recent studies reveal that itaconate inhibits TET2 activity in tumor-associated macrophages within the nasopharyngeal carcinoma (NPC) microenvironment, inducing polarization toward an immunosuppressive M2 phenotype. This reduces phagocytic capacity and impairs CD8^+^ T cell cytotoxic function, this metabolic-epigenetic remodeling promotes immune evasion, proliferation, and invasive behavior of NPC cells, suggesting that targeting the IRG1/ITA/TET2 pathway or restoring TET2 function may provide novel immunotherapeutic strategies for NPC (76). Itaconate regulates inflammation through a multi-layered network, involving TET2 modification, Nrf2 signaling, and ATF3/IκBζ regulation. By inhibiting TET2 activity, itaconate mitigates excessive inflammation. However, in tumor microenvironments, itaconate’s inhibition of TET2 can promote immune evasion, suggesting that targeting this pathway could offer novel strategies for cancer immunotherapy.

### 3.5 Itaconate as a glycolytic inhibitor

GAPDH is a key rate-limiting enzyme in glycolysis, catalyzing the conversion of glyceraldehyde-3-phosphate to 1, 3-bisphosphoglycerate, requiring NAD^+^ and inorganic phosphate. As early as 1981, Emile et al. demonstrated that itaconate inhibits fructose-6-phosphate 2-kinase, reducing fructose-2, 6-bisphosphate production (64). This suggests it may inhibit glycolysis in LPS-activated macrophages, thus suppressing inflammation and oxidative stress (64). Recent studies further confirm that itaconate and its derivatives regulate the glycolytic pathway and exert anti-inflammatory effects (77). Furthermore, alkylation of cysteine residues were detected in several glycolysis-related enzymes, including aldolase A (ALDOA), glyceraldehyde-3-phosphate dehydrogenase (GAPDH), and lactate dehydrogenase A (LDHA) (77, 78).

Mechanistically, itaconate covalently modifies a key cysteine residue, cys22, in GAPDH, inhibiting its enzymatic activity (18). This reduces downstream ALDOA activity and lactate production. In contrast, in Irg1^-^/^-^ macrophages, elevated ALDOA activity, lactate production, and enhanced ECAR were observed, suggesting that itaconate mediates its anti-inflammatory effects by inhibiting glycolysis (18). Notably, despite its broad scope of action, itaconate is produced exclusively in activated macrophages, suggesting it may regulate host-pathogen interactions by altering the local metabolic microenvironment rather than directly targeting bacteria (79). Furthermore, its predominant synthesis within activated macrophages suggests potential enrichment in specific subcellular compartments. When macrophages confine pathogens to these microenvironments, altered metabolic conditions reprogram bacterial metabolic pathways, enabling itaconate’s antibacterial effects (80). In oncology, studies show that 4-octyl itaconate (4-OI) inhibits glycolysis and enhances cuproptosis sensitivity in colorectal cancer cells (81), further supporting its regulatory role in glycolysis while providing a potential new therapeutic strategy and theoretical basis for colorectal cancer treatment (82). Itaconate inhibits glycolysis in macrophages by modifying GAPDH and other enzymes, reducing lactate production and inflammation. This metabolic regulation suggests itaconate can influence host-pathogen interactions and may offer a novel cancer treatment approach, enhancing sensitivity to therapies like cuproptosis in colorectal cancer.

### 3.6 Key variables determining the functional outcome of itaconate

Concentration and Timing. Accumulating evidence indicates that the immunoregulatory effects of itaconate are tightly linked to both its intracellular concentration and the temporal pattern of its induction. During early or acute inflammatory responses, moderate elevations of itaconate driven by IRG1 induction function primarily as a negative-feedback mechanism, limiting SDH activity, restraining succinate oxidation, and reducing mitochondrial ROS–HIF-1α–IL-1β signaling (35). In contrast, sustained itaconate accumulation under conditions of prolonged inflammation or within the tumor microenvironment may shift this protective program toward immune tolerance or immunosuppression (83). Notably, experimental studies with itaconate derivatives suggest that excessive or prolonged exposure may paradoxically enhance IL-1β production or promote inflammatory cell death, highlighting dose- and time-dependent thresholds (84). These observations highlight concentration and timing as central variables shaping the direction of itaconate-mediated immune regulation.

Cellular Specificity. The functional consequences of itaconate signaling also depend on the cellular context in which it is engaged. Although activated macrophages represent the principal source and primary targets of itaconate, distinct macrophage subsets display divergent metabolic states and effector functions. In classically activated inflammatory macrophages, itaconate predominantly suppresses excessive cytokine production and oxidative stress, contributing to inflammation resolution (83). By contrast, in tumor-associated macrophages, persistent itaconate signaling has been linked to metabolic and epigenetic reprogramming that favors immunosuppressive phenotypes and impairs antigen presentation (84). In addition, growing evidence indicates that macrophage-derived itaconate can indirectly influence dendritic cell function and T cell responses through metabolic and cytokine-mediated crosstalk. Together, these findings emphasize that cellular specificity critically determines whether itaconate signaling promotes immune control or immune evasion.

Metabolic Context. Beyond cell type, the broader metabolic environment strongly influences how itaconate-regulated pathways are integrated. Factors such as mitochondrial activity, glycolytic flux, oxygen availability, and substrate supply determine the relative engagement of SDH inhibition, succinate accumulation, and glycolytic suppression (35). In metabolically stressed settings, including hypoxic inflamed tissues and the tumor microenvironment, these conditions can amplify the immunosuppressive consequences of sustained itaconate signaling. Conversely, during acute inflammatory stress, transient metabolic reprogramming driven by itaconate may primarily function to restore redox balance and limit tissue damage. Thus, itaconate acts within a dynamic metabolic landscape, and its immunological effects cannot be dissociated from the surrounding metabolic context.

Molecular Switches. At the molecular level, multiple regulatory nodes function as critical switches that govern the downstream effects of itaconate. These include SDH inhibition–succinate–HIF-1α signaling (4, 23), activation of the Keap1–Nrf2 antioxidant pathway (35), inhibition of TET2-dependent epigenetic programs (84), and electrophilic modification of glycolytic enzymes such as GAPDH (85). The relative dominance and coordination of these pathways determine whether pro-inflammatory or anti-inflammatory transcriptional programs prevail. Importantly, these signaling modules do not operate independently but intersect across metabolic, transcriptional, and epigenetic layers, allowing cooperative or antagonistic interactions depending on cellular and environmental conditions. This interconnected network of molecular switches provides a mechanistic basis for the context-dependent and sometimes opposing immunological outcomes of itaconate.

Collectively, the immunomodulatory effects of itaconate can be summarized within two dominant, context-dependent programs: suppression of acute inflammation and immune dampening during chronic inflammation or tumorigenesis. In acute inflammatory settings, such as early infection or endotoxin exposure, itaconate accumulation acts as an intrinsic negative feedback mechanism that limits excessive immune activation. This protective response is mediated through interconnected pathways, including SDH inhibition–driven succinate control, activation of the Keap1–Nrf2 antioxidant axis, TET2-dependent epigenetic restraint of pro-inflammatory gene expression, and ATF3/IκBζ-associated transcriptional suppression. Together, these mechanisms reduce mitochondrial ROS production, attenuate HIF-1α–IL-1β signaling, and promote inflammation resolution, thereby preserving tissue homeostasis.

In contrast, under conditions of sustained inflammation or within the tumor microenvironment, prolonged itaconate signaling may engage a predominantly immunosuppressive program. Persistent Nrf2 activation, chronic TET2 inhibition, and glycolytic suppression in macrophages can promote immune tolerance, M2-like polarization, and impaired antigen presentation. In these contexts, pathways that are protective during acute inflammation may become maladaptive, contributing to immune paralysis or tumor immune evasion. Thus, the IRG1–itaconate axis functions as a context-sensitive immunometabolic switch, with signaling duration, cellular identity, and microenvironmental cues collectively determining its protective or pathological outcomes.

## 4 Therapeutic implications

A growing body of research indicates that the metabolic axis comprising itaconate and its synthase gene IRG1 (ACOD1) holds great potential for disease intervention. Multiple animal experiments and corresponding mouse disease models consistently show that targeting the IRG1–itaconate pathway can alleviate inflammation, remodel immune metabolism, and improve disease outcomes across various pathological scenarios, providing strong evidence for its therapeutic potential. At the pharmacological level, the chemical and biological properties of itaconate—such as its electrophilicity, antioxidant capacity, and ability to induce metabolic reprogramming—also provide important insights for identifying novel sites of action and designing lead compounds. To validate the mechanism, exogenous itaconate supplementation in bone marrow-derived macrophages, combined with control experiments like IRG1 gene knockout, further replicated and amplified the effects observed with prodrug molecules like 4-OI and DI. This supports the critical role of the IRG1–itaconate pathway as an endogenous axis regulating anti-inflammation and immune metabolism. Currently, most research on the clinical potential of itaconate is still at the preclinical stage, with a lack of large-scale human clinical data. Existing studies mainly focus on animal models and *in vitro* experiments, with more clinical data is needed to confirm the changes in itaconate levels in patient bodily fluids. In some cases, such as during inflammation, itaconate levels may fluctuate, but systematic clinical sample studies to validate this are still lacking (**Table 1**).

**Table 1:** Therapeutic effects of itaconate and its derivatives across diverse disease models.

Disease	Model	Species	Agents	Mechanisms	References
Sepsis	LPS-induced lethality	Mice	4-OI	Activation of Nrf2/HO-1 signaling and inhibition of SDH to suppress inflammation.	Mills et al.
	CLP-induced sepsis	Mice	4-OI	Suppression of glycolysis and reduced pro-inflammatory cytokine production.	Liao et al.
	LPS-induced sepsis	Mice	Exogenous itaconate	TET2 inhibition suppresses pro-inflammatory gene expression.	Domínguez-Andrés et al.
IBD	DSS-induced colitis	Mice	DI	Immune modulation reduces intestinal inflammation.	Wang et al.
	DSS-induced colitis	Mice	4-OI	Nrf2/HO-1 activation protects intestinal barrier and reduces oxidative stress.	Yang et al.
	DSS-induced colitis	Mice	Exogenous itaconate	Inhibition of NF-κB signaling reduces inflammation.	Kim et al.
I/R injury	Myocardial I/R	Mice	DI	SDH inhibition reduces mitochondrial ROS and protects the heart.	Lampropoulou et al.
	Cerebral I/R	Mice	Exogenous itaconate	Nrf2 activation maintains redox balance and reduces damage.	Cordes et al.
	Hepatic I/R	Mice	4-OI	Nrf2/HO-1 activation suppresses NLRP3 inflammasome and liver injury.	Zhang et al.
SLE	PBMCs from SLE patients	Human	4-OI	Nrf2 activation reduces oxidative stress and immune activation.	Tang et al.
	Murine lupus	Mice	4-OI	Nrf2 signaling reduces autoimmune responses and inflammation.	Blanco et al.
MS	EAE	Mice	DI	Nrf2/HO-1 signaling and SDH inhibition regulate inflammation.	Kuo et al.
	Mixed glia	Mice/human	DI;4-OI	NLRP3 inflammasome inhibition reduces neuroinflammation.	Hoyle et al.
CAPS	Cells from CAPS patients	Human	4-OI	NLRP3 inflammasome inhibition.	Hooftman et al.
AAA	AngII-induced AAA	Mice/human	4-OI/DI	Nrf2 activation reduces vascular inflammation and prevents aneurysm progression.	Song et al.
Mastitis	LPS-induced mastitis	Mice	DI	Nrf2 activation suppresses pro-inflammatory cytokines.	Zhao et al.
Endometritis	LPS-induced endometritis	Mice	DI	Nrf2/HO-1 activation inhibits NF-κB.	Xu et al.
Renal fibrosis	Adenine-induced fibrosis	Rats	4-OI	Nrf2 activation reduces oxidative stress and inhibits fibrosis.	Tian et al.
RA	RANKL-induced osteoclastogenesis	Mice	4-OI	TET2 inhibition suppresses osteoclast differentiation and bone resorption.	Sun et al.
	Collagen-induced arthritis	Mice	Exogenous itaconate	NF-κB suppression reduces joint inflammation.	Li et al.
Pulmonary fibrosis	BLM-induced fibrosis	Mice	Inhaled itaconate	Nrf2 activation suppresses fibrosis and oxidative stress.	Haan et al.
	TGF-β1 fibroblast activation	Mice	DI	Inhibition of EMT via Nrf2 signaling.	Han et al.
	BLM-induced fibrosis	Mice	4-OI	Anti-EMT properties reduce pulmonary fibrosis development.’	Wu et al.
Cancer	TAMs (multiple models)	Mice	Endogenous itaconate	TET2 inhibition promotes immune suppression and tumor escape.	Wang et al.
	DEN-induced HCC	Rats	DI	GAPDH and SDH inhibition reduce tumor growth and promote tumor cell death.	Chen et al.
	Colorectal cancer	Human	4-OI	Glycolysis inhibition enhances cuproptosis and cell death in tumors.	Liu et al.
	Nasopharyngeal carcinoma	Mice/human	Endogenous itaconate	TET2 inhibition reprograms macrophages, promoting immune evasion.	Wu et al.
Myogenesis	BaCl2-induced muscle injury	Mice	4-OI	Regulation of SDH and mitochondrial function during muscle regeneration.	Oh et al.

This table summarizes representative preclinical and translational studies evaluating itaconate or its derivatives (e.g., 4-octyl itaconate and dimethyl itaconate) in infectious, autoimmune/inflammatory, ischemia–reperfusion, fibrotic, and cancer-related models. Proposed mechanisms indicate the major pathways reported in each study and are not intended to be exhaustive.

4-OI, 4-octyl itaconate; DI, dimethyl itaconate; LPS, lipopolysaccharide; CLP, cecal ligation and puncture; DSS, dextran sulfate sodium; IBD, inflammatory bowel disease; I/R, ischemia–reperfusion; PBMCs, peripheral blood mononuclear cells; SLE, systemic lupus erythematosus; MS, multiple sclerosis; EAE, experimental autoimmune encephalomyelitis; CAPS, cryopyrin-associated periodic syndromes; AAA, abdominal aortic aneurysm; AngII, angiotensin II; RA, rheumatoid arthritis; RANKL, receptor activator of NF-κB ligand; BLM, bleomycin; EMT, epithelial–mesenchymal transition; TAMs, tumor-associated macrophages; HCC, hepatocellular carcinoma; DEN, diethylnitrosamine; ROS, reactive oxygen species; SDH, succinate dehydrogenase; Nrf2, nuclear factor erythroid 2–related factor 2; HO-1, heme oxygenase-1; TET2, ten-eleven translocation 2; NF-κB, nuclear factor kappa-B; NLRP3, NLR family pyrin domain containing 3; BaCl_2_, barium chloride; GAPDH, glyceraldehyde-3-phosphate dehydrogenase.

### 4.1 Itaconate: a double-edged regulator in sepsis

Sepsis is a systemic response to infection, characterized by a cytokine storm and systemic immune suppression, and can progress to life-threatening dysregulation and multiple-organ failure. Current research indicates that enhancing the IRG1-itaconate axis or using 4-OI in both *in vivo* and *in vitro* experiments can mitigate LPS-induced injury and inflammation, and reduce mortality rates (14, 86, 87).

The mechanisms appear to be multifaceted. Specifically, 4 - OI suppresses Keap1 activity, which in turn activates Nrf2 signaling and attenuates inflammatory responses in mice (42, 43, 86). Other studies suggest 4-OI exerts anti-inflammatory effects in mice by promoting GAPDH alkylation and inhibiting glycolysis. SDH inhibition has also been demonstrated to reduce the release of inflammatory cytokines *in vivo*. Although direct evidence regarding sepsis remains limited, itaconate may likewise mitigate systemic inflammatory responses associated with sepsis by inhibiting SDH activity (23). In an LPS-induced endotoxemia mouse model, exogenous itaconate significantly reduced lung and liver injury and improved survival. This anti-inflammatory/protective effect depended on TET2’s catalytic activity; the effect was absent under TET2-deficient conditions, suggesting the IRG1–itaconate–TET2 axis plays a key regulatory role in sepsis (88). These studies indicate that itaconate’s effects on sepsis likely involve multiple pathways rather than a single target. However, studies suggest that reducing IRG1 and itaconate may represent a strategy to alleviate immunosuppression in sepsis (89). Research on IRG1 and A20 indicates IRG1 may play a significant role in sepsis-associated immunosuppression or immunoparalysis, as both IRG1 and A20 are elevated in peripheral blood mononuclear cells from septic patients (90). Among these, β-glucan, a fungal cell wall component, is known to enhance innate immune function in monocytes by reducing IRG1 expression and thereby reversing tolerance (13). The available evidence is not necessarily conflicting. In sepsis models, moderate activation of the IRG1–itaconate axiscan restrain excessive inflammation through multiple converging mechanisms, including Keap1 alkylation–Nrf2 activation, SDH inhibition with reduced mtROS/HIF-1α–IL-1β signaling, and TET2-dependent epigenetic reprogramming. However, when itaconate is sustained at high levels—or when SDH is over-inhibited—it may tip the host toward immune tolerance/immunoparalysis, increasing vulnerability to secondary infection. Together, these findings suggest that itaconate functions as a dose-, time-, and context-dependent “double-edged” regulator in sepsis. Clinically, this highlights the need to define an effective therapeutic window and to consider stage-specific use, potentially pairing anti-inflammatory benefits early with strategies that avoid or reverse late-phase immunosuppression.

### 4.2 Protective roles of itaconate in ischemia–reperfusion injury

Hepatic ischemia-reperfusion injury (IRI) is a primary cause of liver dysfunction following liver transplantation, hepatectomy, and hemorrhagic shock. Its core mechanism involves an initial interruption of blood flow followed by reperfusion, which triggers excessive reactive oxygen species (ROS) production, disrupts redox homeostasis, and directly damages hepatocytes. ROS also activates Kupffer cells and other cells to release inflammatory mediators, recruiting immune cells and inducing hepatocyte apoptosis (91). In the IRI model—a tissue injury characterized by oxidative stress, inflammatory response, and cell death—the liver possesses an intrinsic antioxidant system that is readily overwhelmed by ROS (92). Notably, itaconate appears to exert protective effects in hepatic IRI. Studies have revealed that in a mouse model of hepatic IRI, IRG1 knockout mice exhibited more severe liver injury than controls, with increased inflammatory mediators like IL-6 and exacerbated inflammatory responses. Supplementation with itaconate derivatives (e.g., 4-OI) effectively mitigated hepatocyte necrosis, reduced ALT/AST levels, and suppressed inflammatory mediator release (91). Mechanistically, itaconate is believed to activate the Nrf2 antioxidant pathway to mitigate oxidative stress and inhibit NLRP3 inflammasome and NF-κB signaling pathways, thereby reducing apoptosis and inflammatory responses (93). As early as 2014, studies demonstrated that dimethylmalonate (DMM) exhibited therapeutic potential for ischemia/reperfusion injury (IRI) in mouse models by inhibiting SDH (94). Because itaconate and derivatives can also inhibit SDH, they may offer similar therapeutic potential in IRI (95). Beyond the liver, itaconate also exhibits protective effects in renal IRI models (96). Research indicates the IRG1-itaconate axis attenuates systemic inflammation and reduces tissue injury following renal ischemia-reperfusion. Furthermore, recent studies suggest itaconate may exert cytoprotective effects in myocardial ischemia-reperfusion injury by modulating metabolic enzymes such as PKM2 (97). Collectively, itaconate shows protective effects across IRI in multiple organs, including the liver, kidneys, and heart, through mechanisms that involve SDH inhibition, Nrf2 activation, and suppression of NLRP3 inflammasome and NF-κB signaling. By modulating these immunometabolic pathways, itaconate may alleviate oxidative stress, dampen inflammation, and reduce cell death, supporting its potential as a metabolic intervention target98. Nevertheless, further studies are required to clarify organ-specific mechanisms and to optimize the route, dosing regimen, and timing of administration.

### 4.3 Roles of itaconate in inflammatory bowel disease

Inflammatory bowel disease primarily encompasses ulcerative colitis (UC) and Crohn’s disease (CD), representing a class of immune-mediated disorders characterized by chronic, intermittent inflammation of the gastrointestinal tract (99). The core pathology of IBD involves activation of inflammatory pathways and inflammasomes (primarily NLRP3) due to intestinal mucosal damage or dysbiosis, leading to massive release of early pro-inflammatory factors such as IL-1β, IL-18, TNF, and IL-6, accompanied by tissue injury (100). Studies have shown increased NF-κB activation in both mucosal epithelial cells and macrophages of IBD patients, leading to the production of TNF-α and other pro-inflammatory factors that exacerbate the inflammatory response (101, 102). Extensive prior research on the effects of itaconate on multiple inflammatory pathways suggests its potential therapeutic impact on IBD (7). Studies in acute colitis mouse models demonstrate that elevating endogenous itaconate or administering 4-OI significantly suppresses disease activity, inhibits intestinal epithelial injury, and reduces proinflammatory factor production during disease flare-ups. Conversely, Acod1/IRG1 knockout mice exhibit heightened susceptibility to colitis, indicating its anti-inflammatory and mucosal protective effects (14, 103). Furthermore, 4-OI can activate Nrf2 by alkylating KEAP1, thereby reducing oxidative stress and downregulating NF-κB-related inflammatory factors. This enhances tight junction proteins, strengthens barrier function, and alleviates colitis symptoms (103–105). Finally, recent studies indicate significant efficacy of 4-OI during the acute phase of IBD, involving modulation of the intestinal barrier, neutrophil migration, and correction of microbiota dysbiosis (64, 106). Overall, itaconate and its derivative 4-OI demonstrate considerable therapeutic potential in acute IBD. This is achieved through mechanisms including Nrf2 pathway activation, reduction of oxidative stress, inhibition of NF-κB signaling, enhancement of intestinal barrier function, and correction of gut microbiota dysbiosis (107). These findings support the potential of itaconate-based interventions as effective therapeutic targets for IBD by fortifying the epithelial barrier and modulating the gut microbiota balance (107).

### 4.4 Itaconate in RA bone destruction

The core pathology of rheumatoid arthritis (RA) involves enhanced bone erosion driven by inflammation-induced excessive osteoclast activation. The interaction of inflammatory mediators such as TNF-α and IL-1β leads to progressive disease deterioration, disrupting bone turnover balance by impairing bone resorption (108–111). Recent studies indicate that administration of itaconate or 4-OI in human samples and mouse models reduces osteoclast numbers/volumes and bone resorption pit areas. *In vivo* experiments confirm that itaconate/4-OI effectively inhibits TET2 activity through reduced joint swelling and inflammation scores, along with histological evidence of diminished bone erosion (108, 112, 113). This mechanism affects the expression of TET2-related genes involved in bone resorption, suppresses excessive osteoclast differentiation and function, and effectively reverses or alleviates bone destruction processes. Previous basic research demonstrated that itaconate directly inhibits TET DNA dioxygenases (primarily TET2), reduces 5hmC at inflammatory gene loci, and limits NF-κB/STAT pro-inflammatory pathways (109). The aforementioned studies extended this mechanism to bone metabolism and completed systematic validation in RA (35, 68, 114). Given RA’s multifactorial pathogenesis, it is plausible that itaconate may also influence RA through other inflammatory pathways. However, excessive or improper derivatives may induce adverse effects or even pro-inflammatory tendencies (98, 115). In chronic inflammation, these risks are particularly worrying, as long-term or improper use may aggravate tissue damage or disrupt immune regulation. In addition, the effectiveness of itaconate should be evaluated in the context of different diseases, especially autoimmune diseases with different inflammatory characteristics. Itaconate and its derivative (4-OI) help reduce bone erosion in rheumatoid arthritis (RA) by inhibiting TET2 activity, decreasing the expression of bone resorption-related genes, and suppressing excessive osteoclast differentiation and function. These findings suggest that itaconate may impact RA progression through various inflammatory pathways. However, improper or excessive use could lead to adverse effects or promote inflammation, highlighting the importance of carefully controlling dosage, administration, and treatment timing. Furthermore, prospective studies are needed to confirm the effectiveness of itaconate when used in combination with anti-TNF/anti-IL-6 agents (116–119).

### 4.5 Itaconate in pulmonary fibrosis

Pulmonary fibrosis (PF) is a chronic progressive disease caused by multiple etiologies, characterized by excessive repair of lung tissue and deposition of extracellular matrix (ECM) (120). When the lungs are exposed to external stimuli, pulmonary epithelial cells and macrophages are activated, releasing large amounts of inflammatory cytokines that trigger localized inflammatory responses (121, 122). In a 2020 study, researchers detected reduced itaconate levels and decreased ACOD1 (IRG1) expression in bronchoalveolar lavage fluid (BAL) and airway macrophages (AM) from pulmonary fibrosis patients (122). Subsequently, in a bleomycin (BLM)-induced pulmonary fibrosis (PF) mouse model, mice were grouped by RF stage. Comparing the Acod1 knockout group with the control group and administering inhaled itaconate intervention revealed that the knockout group exhibited persistent pulmonary fibrosis. The inhalation of itaconate alleviated pulmonary fibrosis and reduced associated inflammatory responses, demonstrating its potential therapeutic efficacy (122). Subsequently, HAN et al. induced fibroblast differentiation with TGF-β1 and treated RF mice with dimethyl itaconate (DMI). They found DMI activated NRF2, downregulated TXNIP, inhibited TGF-β1-induced FMD and ROS production, and effectively alleviated pulmonary fibrosis phenotypes in mice (123, 124). Wu et al. demonstrated that 4-OI significantly reduced pulmonary fibrosis and collagen deposition by inhibiting macrophage-mediated epithelial-mesenchymal transition through NRF2 activation (125). Itaconate and its derivatives, including 4-OI and DMI, demonstrate potential therapeutic effects in pulmonary fibrosis by targeting key inflammatory pathways and activating NRF2. Through modulating epithelial-mesenchymal transition (EMT), reducing collagen deposition, and enhancing lung function, itaconate provides a promising new treatment approach for this debilitating disease (125, 126). However, further research is needed to fully elucidate the underlying mechanisms and optimize its clinical applications.

### 4.6 The therapeutic effect of itaconate in tumors

Although the immunoregulatory role of itaconate in inflammation has been extensively studied, recent research has increasingly suggested that itaconate promotes tumor progression by reshaping the tumor microenvironment (TME) through its effects on cancer’s immunometabolic pathways (127, 128). As early as 2018, researchers established intraperitoneal/peritoneal tumor models by injecting tumor cells (such as B16 melanoma cells or ID8 ovarian cancer cells) into the abdominal cavity of mice, from which they isolated peritoneal tissue-resident macrophages (pResMφ). Metabolomic analysis revealed significant metabolic reprogramming in these macrophages, characterized by enhanced fatty acid oxidation and oxidative phosphorylation (OXPHOS) levels, accompanied by markedly elevated intracellular itaconate content. Additionally, they observed significantly increased IRG1 levels in the monocytes isolated from the ascites of ovarian cancer patients. Further studies revealed that targeting the IRG1–itaconate pathway effectively suppressed tumor volume growth in mouse models, suggesting the therapeutic potential for this metabolic axis in metastatic tumor microenvironments (129). In hepatocellular carcinoma cells, previous research confirmed that in IRG1 knockout mice, tumor node ratio, liver-to-body weight ratio, and *in vivo* tumor volume—key tumor burden indicators—were significantly reduced. Irg1 knockout mice exhibited slowed tumor progression accompanied by significantly reduced CD8^+^ T cell infiltration, suggesting suppression of antitumor immunity. Targeting IRG1/fumarate may represent a novel immunotherapy strategy for HCC, particularly involving CD8^+^ T cells (53, 130). Similarly, in 2024, a mouse model of nasopharyngeal carcinoma (NPC) revealed that tumor-associated macrophages enhance itaconate production by upregulating IRG1. This promotes M2 macrophage polarization, reduces CD8^+^ T cell activity, and facilitates tumor cell growth and immune evasion through TET2-mediated mechanisms (76). Wang et al. reported that DI blocks IL-1β release and reduces macrophage infiltration into the tumor microenvironment, thereby alleviating excessive inflammation in colitis and lowering the risk of colitis-associated colorectal cancer. However, recent studies suggest that itaconate may conversely drive a pro-tumor phenotype by reshaping macrophage metabolism (131). Similarly, studies have found that IRG1 is upregulated in both clinical specimens and human glioma cell lines, with poor prognosis associated with high IRG1 expression (89). Furthermore, research on itaconate extends beyond the compound itself. Studies indicate that colorectal cancer actively acquires itaconate via the transporter SLC13A3, alkylating PD-L1 at the Cys272 site to inhibit its ubiquitination and degradation, thereby enhancing resistance to PD-L1 monoclonal antibodies. Targeting the SLC13A3 protein effectively alleviates or even reverses PD-L1 resistance (132, 133). In 2022, clinical evaluations commenced in hepatocellular carcinoma. As an anticancer agent, DI was administered via intraperitoneal injection of DEN (diethylnitrosamine) to induce hepatocellular carcinoma in albino Wistar rats (134). Results showed tumor nodule shrinkage and normalized liver structure in the treatment group compared to controls, indicating itaconate induces cancer cell death (135, 136). Recent studies reveal that certain tumor cells can endogenously synthesize itaconate by upregulating related genes (135). This tumor-derived itaconate not only fails to suppress immunity but also enhances tumor immunogenicity, thereby boosting immunotherapy efficacy. This finding contrasts sharply with the traditional perception of itaconate as a pro-tumor agent. Therefore, we hypothesize that as a key intermediate metabolite linking inflammation and metabolism, itaconate may exert a classic “double-edged sword” effect in tumors. On one hand, IRG1 is induced to high expression in myeloid cells such as tumor-associated macrophages. There, it suppresses SDH activity and activates multiple anti-inflammatory signaling pathways, thereby weakening antigen presentation capacity and effector T cell function. This shapes an immunosuppressive microenvironment that promotes tumor immune escape. Conversely, within certain tumor cells, the IRG1–itaconate axis enhancing antigen processing and MHC-I molecule expression, increasing tumor cell immunogenicity and sensitivity to immunotherapy. This suggests its biological effects exhibit significant cell-type and context-dependent properties.

In summary, itaconate exerts inconsistent effects on different tumor-associated cells, and exogenous versus endogenous itaconate may also influence tumor cells differently. Overall, the role of itaconate in tumors is dual. By regulating immune metabolic pathways in the tumor microenvironment, it can not only promote immune escape but also enhance the immunogenicity of tumor cells (135). Its biological effects are significantly influenced by concentration, time and microenvironment. Therefore, itaconate has the potential to become an important target for tumor immunometabolic therapy and is worthy of further exploration in future pharmacological research and clinical translation.

### 4.7 Disease-specific mechanism comparison

Although the core molecular targets of itaconate are largely conserved, its immunological outcomes differ substantially across disease contexts. These differences reflect variations in inflammatory duration, dominant immune cell populations, metabolic states, and local microenvironmental conditions. Among the diverse pathological settings discussed in this review, sepsis, inflammatory bowel disease (IBD), and cancer represent three representative scenarios that illustrate how the IRG1–itaconate axis exerts distinct context-dependent functions (7, 117).

In sepsis, particularly during the acute phase of systemic inflammation, itaconate primarily functions as a protective negative-feedback regulator (95). Rapid induction of IRG1 in activated macrophages leads to elevated intracellular itaconate levels, which inhibit SDH activity, limit succinate-driven mitochondrial ROS production, and attenuate HIF-1α–IL-1β signaling (86, 117). In parallel, activation of the Keap1–Nrf2 pathway and TET2-dependent epigenetic restraint further restrict excessive pro-inflammatory gene expression (11). In this setting, the anti-inflammatory effects of itaconate are closely linked to the need for timely control of cytokine overproduction and prevention of tissue damage.

In inflammatory bowel disease, the role of itaconate is more complex and strongly influenced by disease stage and tissue context. On the one hand, itaconate-mediated regulation of macrophage activation and the ATF3/IκBζ axis contributes to limiting intestinal inflammation and supporting epithelial barrier integrity (98, 117). On the other hand, sustained SDH inhibition and succinate accumulation can reshape the local metabolic environment and influence gut microbial composition, potentially favoring opportunistic pathogens over short-chain fatty acid–producing commensals. Thus, in IBD, the net effect of itaconate signaling reflects a balance between immune regulation and host–microbiota interactions, with local metabolic conditions serving as key determinants.

In cancer, particularly within the tumor microenvironment, prolonged activation of the IRG1–itaconate axis often shifts toward an immunosuppressive profile. Tumor-associated macrophages exhibit sustained itaconate accumulation, which promotes chronic inhibition of TET2, persistent Nrf2 activation, and suppression of glycolytic metabolism (128). These changes favor polarization toward an M2-like phenotype, impair antigen presentation, and reduce cytotoxic T cell activity, thereby facilitating immune evasion. In this context, metabolic pathways that limit inflammation during acute immune responses may instead support tumor progression when chronically engaged.

Taken together, these disease-specific comparisons highlight that the biological effects of itaconate are neither uniformly beneficial nor detrimental. Rather, they are determined by the pathological context, including the duration of inflammatory signaling, cellular composition, metabolic state, and microenvironmental cues. Recognizing this context dependency is essential for understanding the dual roles of the IRG1–itaconate axis and for guiding its potential therapeutic exploitation (117).

### 4.8 Translational challenges

Although itaconate has demonstrated significant immunomodulatory potential in preclinical studies, several key challenges must be addressed before its clinical application. First, the immunomodulatory effects of itaconate need to be precisely targeted at specific cell types, especially tumor-associated macrophages (TAMs) (130, 135). To ensure therapeutic efficacy and minimize side effects, targeted delivery systems must be developed and optimized to efficiently and accurately deliver the drug to the intended cells. There is still much room for improvement in existing delivery systems, requiring further research and technological advancements. Secondly, itaconate exhibits dual effects of anti-inflammatory and immune suppression. A key challenge is how to control its dose and time-dependent effects to ensure it remains within an effective range, particularly as excessive doses could lead to over-suppression of the immune system, triggering immune paralysis, especially in diseases such as sepsis (5, 7). Therefore, determining the optimal therapeutic window and precisely regulating the dosage and timing to avoid adverse reactions remains a critical issue. Lastly, the long-term safety of itaconate, especially in the treatment of chronic diseases, remains unclear. Prolonged use could pose potential risks, particularly concerning the immune system (127). Therefore, before clinical application, comprehensive evaluations of its long-term safety are required, alongside the development of effective monitoring methods to ensure itaconate levels remain within a safe and effective range.

## 5 Itaconate and immune cells

Itaconate, an important immunometabolic compound generated by immune-responsive gene 1 (IRG1), which encodes aconitate decarboxylase 1 (ACOD1) in the tricarboxylic acid (TCA) cycle, not only participates in signal transduction and metabolic reprogramming within tumor cells but also contributes to the functional remodeling of various immune cells, including macrophages, neutrophils, and dendritic cells (7, 62, 98). Macrophages serve as the primary source of the itaconate (**Figure 1**). In macrophages, IRG1 is significantly upregulated under inflammatory stimulation, promoting itaconate accumulation. This acid inhibits M1 proinflammatory responses in macrophages, induces a shift toward the M2 phenotype, and reduces plaque inflammation in disease models such as atherosclerosis (62). Within the immune microenvironment, high levels of itaconate in macrophages drive Eomes-associated epigenetic programs that induce CD8^+^ T cell exhaustion, upregulate inhibitory receptors such as PD-1, TIM-3 and promote tumor progression in hepatocellular carcinoma and other cancers (53). In neutrophils, studies indicate that Mycoplasma pneumoniae infection induces IRG1 expression and itaconate production. This metabolite impairs neutrophil bactericidal activity by inhibiting SDH activity and reducing mitochondrial ROS production, thereby facilitating bacterial survival and tissue injury (137). Neutrophil-derived extracellular vesicles (NDEVs) enriched in miR-27a-3p inhibit the expression of Suclg1, a mitochondrial enzyme involved in itaconate catabolism, thereby reducing its degradation and promoting itaconate accumulation within macrophages, further reshaping their inflammatory phenotype (138). In dendritic cells, pathogen- or tumor-associated stimuli drive itaconate production. On one hand, during Plasmodium falciparum infection, it enhances mtDNA-mediated PD-L1 expression to suppress maturation of monocyte-derived DCs and T cell responses (139). while in tumor models and mRNA cancer vaccines, itaconate derived from lymph node or tumor-resident macrophages similarly impairs antigen presentation and cross-sensitization capabilities of DCs, reducing specific CD8^+^ T cell activation and diminishing the therapeutic effects of anti-PD-1 and mRNA vaccines (140, 141). Collectively, itaconate is no longer viewed solely as a macrophage metabolic marker but rather as a pivotal metabolic hub linking metabolic reprogramming to functional remodeling across diverse immune cells. It establishes a critical “metabolism-inflammation-effect” pathway that bridges innate and adaptive immunity, offering novel insights for understanding its double-edged role in inflammation and tumors from a cellular lineage perspective. However, the clinical application of itaconate faces several challenges, including the optimization of targeted delivery systems, precise dose regulation, and long-term safety evaluation. These issues require further research and technological advancements to be effectively addressed.

## 6 Conclusion

As an endogenous metabolite originating from the tricarboxylic acid cycle, itaconate has garnered significant attention in recent years due to its multifaceted roles in immunomodulation, metabolic reprogramming, and inflammation control. Based on its broad anti-inflammatory mechanisms and the therapeutic effects demonstrated by exogenous itaconate and its derivatives *in vivo*, itaconate holds promise as an alternative therapeutic strategy for immune-inflammatory diseases. At appropriate doses, itaconate demonstrates significant anti-inflammatory effects; however, excessive accumulation may lead to immunosuppression or immune paralysis. the dual effects of itaconate suggest that its role in different diseases is influenced by factors such as dose, timing, and delivery method, making precise dosing and targeted regulation particularly important Therefore, its effects in different diseases are context-dependent, suggesting that translational applications require careful balancing between anti-inflammatory benefits and anti-infective/anti-tumor capabilities. Numerous questions remain regarding itaconate, including the existence of specific receptors and extracellular transport mechanisms, the precise effects of different cystine adducts on protein function, and the definition of the efficacy and safety window based on dose-timing-delivery. Overall, whether by enhancing the endogenous IRG1–itaconate axis or developing more controllable, selective derivatives, itaconate holds promise as a strategic molecule bridging metabolic reprogramming and immune regulation. Future translational research should focus on targeted delivery systems, derivative optimization, and enhancing the selectivity and control of itaconate, advancing its therapeutic application in inflammatory diseases and certain infectious diseases.

## References

[1] Nair S Huynh JP Lampropoulou V Loginicheva E Esaulova E Gounder AP . Irg1 expression in myeloid cells prevents immunopathology during M. Tuberculosis Infection J Exp Med. (2018) 215:1035–45. doi: 10.1084/jem.20180118, PMID: 29511063 PMC5881474

[2] Michelucci A Cordes T Ghelfi J Pailot A Reiling N Goldmann O . Immune-responsive gene 1 protein links metabolism to immunity by catalyzing itaconic acid production. Proc Natl Acad Sci USA. (2013) 110:7820–5. doi: 10.1073/pnas.1218599110, PMID: 23610393 PMC3651434

[3] O’Neill LAJ Artyomov MN . Itaconate: the poster child of metabolic reprogramming in macrophage function. Nat Rev Immunol. (2019) 19:273–81. doi: 10.1038/s41577-019-0128-5, PMID: 30705422

[4] Mills EL Kelly B Logan A Costa ASH Varma M Bryant CE . Succinate dehydrogenase supports metabolic repurposing of mitochondria to drive inflammatory macrophages. Cell. (2016) 167:457–470.e13. doi: 10.1016/j.cell.2016.08.064, PMID: 27667687 PMC5863951

[5] Yang W Wang Y Tao K Li R . Metabolite itaconate in host immunoregulation and defense. Cell Mol Biol Lett. (2023) 28:100. doi: 10.1186/s11658-023-00503-3, PMID: 38042791 PMC10693715

[6] Shi J Cai C . Research progress on the mechanism of itaconate regulating macrophage immunometabolism. Front Immunol. (2022) 13:937247. doi: 10.3389/fimmu.2022.937247, PMID: 35812373 PMC9259868

[7] Peace CG O’Neill LAJ . The role of itaconate in host defense and inflammation. J Clin Invest. (2022) 132:e148548. doi: 10.1172/JCI148548, PMID: 35040439 PMC8759771

[8] Shin J-H Yang J-Y Jeon B-Y Yoon YJ Cho S-N Kang Y-H . (1)H NMR-based metabolomic profiling in mice infected with mycobacterium tuberculosis. J Proteome Res. (2011) 10:2238–47. doi: 10.1021/pr101054m, PMID: 21452902

[9] Strelko CL Lu W Dufort FJ Seyfried TN Chiles TC Rabinowitz JD . Itaconic acid is a mammalian metabolite induced during macrophage activation. J Am Chem Soc. (2011) 133:16386–9. doi: 10.1021/ja2070889, PMID: 21919507 PMC3216473

[10] Zhu L Chen L . Progress in research on paclitaxel and tumor immunotherapy. Cell Mol Biol Lett. (2019) 24:40. doi: 10.1186/s11658-019-0164-y, PMID: 31223315 PMC6567594

[11] Mills EL Ryan DG Prag HA Dikovskaya D Menon D Zaslona Z . Itaconate is an anti-inflammatory metabolite that activates nrf2 via alkylation of KEAP1. Nature. (2018) 556:113–7. doi: 10.1038/nature25986, PMID: 29590092 PMC6047741

[12] Marques E Kramer R Ryan DG . Multifaceted mitochondria in innate immunity. NPJ Metab Health Dis. (2024) 2:6. doi: 10.1038/s44324-024-00008-3, PMID: 38812744 PMC11129950

[13] Domínguez-Andrés J Novakovic B Li Y Scicluna BP Gresnigt MS Arts RJW . The itaconate pathway is a central regulatory node linking innate immune tolerance and trained immunity. Cell Metab. (2019) 29:211–220.e5. doi: 10.1016/j.cmet.2018.09.003, PMID: 30293776

[14] Yang W Wang Y Wang T Li C Shi L Zhang P . Protective effects of IRG1/itaconate on acute colitis through the inhibition of gasdermins-mediated pyroptosis and inflammation response. Genes Dis. (2023) 10:1552–63. doi: 10.1016/j.gendis.2022.05.039, PMID: 37397544 PMC10311025

[15] Freitas AC Rodrigues D Rocha-Santos TAP Gomes AMP Duarte AC . Marine biotechnology advances towards applications in new functional foods. Biotechnol Adv. (2012) 30:1506–15. doi: 10.1016/j.bioteChadv.2012.03.006, PMID: 22484300

[16] Luan HH Medzhitov R . Food fight: role of itaconate and other metabolites in antimicrobial defense. Cell Metab. (2016) 24:379–87. doi: 10.1016/j.cmet.2016.08.013, PMID: 27626199 PMC5024735

[17] Qian W-Z Ou L Li C-X Pan J Xu J-H Chen Q . Evolution of glucose dehydrogenase for cofactor regeneration in bioredox processes with denaturing agents. ChemBioChem. (2020) 21:2680–8. doi: 10.1002/cbic.202000196, PMID: 32324965

[18] Liao S-T Han C Xu D-Q Fu X-W Wang J-S Kong L-Y . 4-octyl itaconate inhibits aerobic glycolysis by targeting GAPDH to exert anti-inflammatory effects. Nat Commun. (2019) 10:5091. doi: 10.1038/s41467-019-13078-5, PMID: 31704924 PMC6841710

[19] Maciejczyk M Heropolitanska-Pliszka E Pietrucha B Sawicka-Powierza J Bernatowska E Wolska-Kusnierz B . Antioxidant defense redox homeostasis and oxidative damage in children with ataxia telangiectasia and nijmegen breakage syndrome. Front Immunol. (2019) 10:2322. doi: 10.3389/fimmu.2019.02322, PMID: 31611883 PMC6776633

[20] Cordes T Wallace M Michelucci A Divakaruni AS Sapcariu SC Sousa C . Immunoresponsive gene 1 and itaconate inhibit succinate dehydrogenase to modulate intracellular succinate levels *. J Biol Chem. (2016) 291:14274–84. doi: 10.1074/jbc.M115.685792, PMID: 27189937 PMC4933182

[21] Martínez-Reyes I Chandel NS . Mitochondrial TCA cycle metabolites control physiology and disease. Nat Commun. (2020) 11:102. doi: 10.1038/s41467-019-13668-3, PMID: 31900386 PMC6941980

[22] Bauernfeind F Ablasser A Bartok E Kim S Schmid-Burgk J Cavlar T . Inflammasomes: current understanding and open questions. Cell Mol Life Sci. (2011) 68:765–83. doi: 10.1007/s00018-010-0567-4, PMID: 21072676 PMC11114650

[23] Lampropoulou V Sergushichev A Bambouskova M Nair S Vincent EE Loginicheva E . Itaconate links inhibition of succinate dehydrogenase with macrophage metabolic remodeling and regulation of inflammation. Cell Metab. (2016) 24:158–66. doi: 10.1016/j.cmet.2016.06.004, PMID: 27374498 PMC5108454

[24] Tannahill GM Curtis AM Adamik J Palsson-McDermott EM McGettrick AF Goel G . Succinate is an inflammatory signal that induces IL-1β through HIF-1α. Nature. (2013) 496:238–42. doi: 10.1038/nature11986, PMID: 23535595 PMC4031686

[25] Dai M Bu S Miao Z . Succinate metabolism: underlying biological mechanisms and emerging therapeutic targets in inflammatory bowel disease. Front Immunol. (2025) 16:1630310. doi: 10.3389/fimmu.2025.1630310, PMID: 41000375 PMC12457111

[26] Fernández-Veledo S Vendrell J . Gut microbiota-derived succinate: friend or foe in human metabolic diseases? Rev Endocr Metab Disord. (2019) 20:439–47. doi: 10.1007/s11154-019-09513-z, PMID: 31654259 PMC6938788

[27] Kokkinias K Sabag-Daigle A Kim Y Leleiwi I Shaffer M Kevorkian R . Time-resolved multi-omics reveals diverse metabolic strategies of salmonella during diet-induced inflammation. mSphere. (2024) 9:e00534–24. doi: 10.1128/msphere.00534-24, PMID: 39254340 PMC11520297

[28] Zhang S Morgan X Dogan B Martin F-P Strickler S Oka A . Mucosal metabolites fuel the growth and virulence of E. Coli linked to crohn’s disease. JCI Insight. (2022) 7:e157013. doi: 10.1172/jci.insight.157013, PMID: 35413017 PMC9220930

[29] Spiga L Winter MG Furtado De Carvalho T Zhu W Hughes ER Gillis CC . An oxidative central metabolism enables salmonella to utilize microbiota-derived succinate. Cell Host Microbe. (2017) 22:291–301.e6. doi: 10.1016/j.chom.2017.07.018, PMID: 28844888 PMC5599368

[30] Connors J Dawe N Van Limbergen J . The role of succinate in the regulation of intestinal inflammation. Nutrients. (2018) 11:25. doi: 10.3390/nu11010025, PMID: 30583500 PMC6356305

[31] Abed DA Goldstein M Albanyan H Jin H Hu L . Discovery of direct inhibitors of keap1–nrf2 protein–protein interaction as potential therapeutic and preventive agents. Acta Pharm Sin B. (2015) 5:285–99. doi: 10.1016/j.apsb.2015.05.008, PMID: 26579458 PMC4629420

[32] Liu S Pi J Zhang Q . Signal amplification in the KEAP1-NRF2-ARE antioxidant response pathway. Redox Biol. (2022) 54:102389. doi: 10.1016/j.redox.2022.102389, PMID: 35792437 PMC9287733

[33] Bambouskova M Gorvel L Lampropoulou V Sergushichev A Loginicheva E Johnson K . Electrophilic properties of itaconate and derivatives regulate the IκBζ–ATF3 inflammatory axis. Nature. (2018) 556:501–4. doi: 10.1038/s41586-018-0052-z, PMID: 29670287 PMC6037913

[34] Baird L Yamamoto M . The molecular mechanisms regulating the KEAP1-NRF2 pathway. Mol Cell Biol. (2020) 40:e00099–20. doi: 10.1128/MCB.00099-20, PMID: 32284348 PMC7296212

[35] Pålsson-McDermott EM O’Neill LAJ . Gang of 3: how the krebs cycle-linked metabolites itaconate succinate and fumarate regulate macrophages and inflammation. Cell Metab. (2025) 37:1049–59. doi: 10.1016/j.cmet.2025.03.004, PMID: 40169002

[36] Otsuki A Yamamoto M . Cis-element architecture of nrf2–sMaf heterodimer binding sites and its relation to diseases. Arch Pharmacal Res. (2020) 43:275–85. doi: 10.1007/s12272-019-01193-2, PMID: 31792803

[37] Kimura M Yamamoto T Zhang J Itoh K Kyo M Kamiya T . Molecular basis distinguishing the DNA binding profile of nrf2-maf heterodimer from that of maf homodimer. J Biol Chem. (2007) 282:33681–90. doi: 10.1074/jbc.M706863200, PMID: 17875642

[38] Hirotsu Y Katsuoka F Funayama R Nagashima T Nishida Y Nakayama K . Nrf2–mafG heterodimers contribute globally to antioxidant and metabolic networks. Nucleic Acids Res. (2012) 40:10228–39. doi: 10.1093/nar/gks827, PMID: 22965115 PMC3488259

[39] Dinkova-Kostova AT Kostov RV Canning P . Keap1 the cysteine-based mammalian intracellular sensor for electrophiles and oxidants. Arch Biochem Biophysics. (2017) 617:84–93. doi: 10.1016/j.abb.2016.08.005, PMID: 27497696 PMC5339396

[40] Eggler AL Liu G Pezzuto JM van Breemen RB Mesecar AD . Modifying specific cysteines of the electrophile-sensing human keap1 protein is insufficient to disrupt binding to the nrf2 domain neH2. Proc Natl Acad Sci. (2005) 102:10070–5. doi: 10.1073/pnas.0502402102, PMID: 16006525 PMC1177374

[41] Rachakonda G Xiong Y Sekhar KR Stamer SL Liebler DC Freeman ML . Covalent modification at cys151 dissociates the electrophile sensor keap1 from the ubiquitin ligase CUL3. Chem Res Toxicol. (2008) 21:705–10. doi: 10.1021/tx700302s, PMID: 18251510

[42] Kobayashi EH Suzuki T Funayama R Nagashima T Hayashi M Sekine H . Nrf2 suppresses macrophage inflammatory response by blocking proinflammatory cytokine transcription. Nat Commun. (2016) 7:11624. doi: 10.1038/ncomms11624, PMID: 27211851 PMC4879264

[43] Vomund S Schäfer A Parnham M Brüne B Von Knethen A . Nrf2 the master regulator of anti-oxidative responses. IJMS. (2017) 18:2772. doi: 10.3390/ijms18122772, PMID: 29261130 PMC5751370

[44] Saha S Buttari B Panieri E Profumo E Saso L . An overview of nrf2 signaling pathway and its role in inflammation. Molecules. (2020) 25:5474. doi: 10.3390/molecules25225474, PMID: 33238435 PMC7700122

[45] Ngo V Duennwald ML . Nrf2 and oxidative stress: A general overview of mechanisms and implications in human disease. Antioxidants. (2022) 11:2345. doi: 10.3390/antiox11122345, PMID: 36552553 PMC9774434

[46] Shen S Li J Wei Z Liu Y Kang L Gu R . Immune-response gene 1 deficiency aggravates inflammation-triggered cardiac dysfunction by inducing M1 macrophage polarization and aggravating ly6Chigh monocyte recruitment. Biol Direct. (2024) 19:86. doi: 10.1186/s13062-024-00521-x, PMID: 39350193 PMC11441264

[47] Zhang T Li K Qiu J Zhang L Wang X Zhang Q . Irg1/itaconate activates nrf2/HO-1 pathway and mitigates septic liver injury in mice. Eur J Inflammation. (2024) 22:1721727X241241360. doi: 10.1177/1721727X241241360

[48] Song J Zhang Y Frieler RA Andren A Wood S Tyrrell DJ . Itaconate suppresses atherosclerosis by activating a nrf2-dependent antiinflammatory response in macrophages in mice. J Clin Invest. (2024) 134:e173034. doi: 10.1172/JCI173034, PMID: 38085578 PMC10849764

[49] Zheng Y Chen Z She C Lin Y Hong Y Shi L . Four-octyl itaconate activates nrf2 cascade to protect osteoblasts from hydrogen peroxide-induced oxidative injury. Cell Death Dis. (2020) 11:772. doi: 10.1038/s41419-020-02987-9, PMID: 32943614 PMC7499214

[50] Yu C Xiao J-H . The keap1-nrf2 system: A mediator between oxidative stress and aging. Oxid Med Cell Longevity. (2021) 2021:6635460. doi: 10.1155/2021/6635460, PMID: 34012501 PMC8106771

[51] Muri J Wolleb H Broz P Carreira EM Kopf M . Electrophilic Nrf2 Activators and Itaconate Inhibit Inflammation at Low Dose and Promote IL-1β Production and Inflammatory Apoptosis at High Dose. Redox Biol. (2020) 36:101647. doi: 10.1016/j.redox.2020.101647, PMID: 32863237 PMC7387846

[52] Yang X Liang B Zhang L Zhang M Ma M Qing L . Ursolic acid inhibits the proliferation of triple−negative breast cancer stem−like cells through NRF2−mediated ferroptosis. Oncol Rep. (2024) 52:94. doi: 10.3892/or.2024.8753, PMID: 38847277 PMC11184361

[53] Gu X Wei H Suo C Shen S Zhu C Chen L . Itaconate promotes hepatocellular carcinoma progression by epigenetic induction of CD8+ T-cell exhaustion. Nat Commun. (2023) 14:8154. doi: 10.1038/s41467-023-43988-4, PMID: 38071226 PMC10710408

[54] Fu Y Liu T He S Zhang Y Tan Y Bai Y . Ursolic acid reduces oxidative stress injury to ameliorate experimental autoimmune myocarditis by activating nrf2/HO-1 signaling pathway. Front Pharmacol. (2023) 14:1189372. doi: 10.3389/fphar.2023.1189372, PMID: 37547335 PMC10403233

[55] Tang C Wang X Xie Y Cai X Yu N Hu Y . 4-octyl itaconate activates nrf2 signaling to inhibit pro-inflammatory cytokine production in peripheral blood mononuclear cells of systemic lupus erythematosus patients. Cell Physiol biochem: Int J Exp Cell Physiol Biochem Pharmacol. (2018) 51:979–90. doi: 10.1159/000495400, PMID: 30466076

[56] Salman S Paulet V Hardonnière K Kerdine-Römer S . The role of NRF2 transcription factor in inflammatory skin diseases. BioFactors. (2025) 51:e70013. doi: 10.1002/biof.70013, PMID: 40207460 PMC11983367

[57] Qin J-J Cheng X-D Zhang J Zhang W-D . Dual roles and therapeutic potential of keap1-nrf2 pathway in pancreatic cancer: A systematic review. Cell Commun Signal. (2019) 17:121. doi: 10.1186/s12964-019-0435-2, PMID: 31511020 PMC6740038

[58] Hai T Hartman MG . The molecular biology and nomenclature of the activating transcription factor/cAMP responsive element binding family of transcription factors: activating transcription factor proteins and homeostasis. Gene. (2001) 273:1–11. doi: 10.1016/S0378-1119(01)00551-0, PMID: 11483355

[59] Kim EY Shin HY Kim JY Kim DG Choi YM Kwon HK . ATF3 plays a key role in Kdo2-lipid A-induced TLR4-dependent gene expression via NF-κB activation. PloS One. (2010) 5:e14181. doi: 10.1371/journal.pone.0014181, PMID: 21152039 PMC2996292

[60] Dempsey LA . Immunoregulatory itaconate. Nat Immunol. (2018) 19:511–1. doi: 10.1038/s41590-018-0123-1, PMID: 29777218

[61] Zhao M Cui Y Wang F Wu F Li C Liu S . Ursolic acid regulates immune balance modulates gut microbial metabolism and improves liver health in mice. IJMS. (2024) 25:10623. doi: 10.3390/ijms251910623, PMID: 39408951 PMC11477038

[62] Kong X Xu L Mou Z Lyu W Shan K Wang L . The anti-inflammatory effects of itaconate and its derivatives in neurological disorders. Cytokine Growth Factor Rev. (2024) 78:37–49. doi: 10.1016/j.cytogfr.2024.07.001, PMID: 38981775

[63] Michaels M Madsen KL . Immunometabolism and microbial metabolites at the gut barrier: lessons for therapeutic intervention in inflammatory bowel disease. Mucosal Immunol. (2023) 16:72–85. doi: 10.1016/j.mucimm.2022.11.001, PMID: 36642380

[64] Kang M Tang D Huang W Zhou M Ma Q Lan Y . Emerging role of itaconate in inflammatory bowel disease: from multitarget immunometabolic mechanisms to therapeutic translation. Int Immunopharmacol. (2025) 166:115632. doi: 10.1016/j.intimp.2025.115632, PMID: 41043316

[65] Zhang X Zhang Y Wang C Wang X . TET (Ten-eleven translocation) family proteins: structure biological functions and applications. Signal Transduction Targeted Ther. (2023) 8:297. doi: 10.1038/s41392-023-01537-x, PMID: 37563110 PMC10415333

[66] Zhang Q Zhao K Shen Q Han Y Gu Y Li X . Tet2 is required to resolve inflammation by recruiting hdac2 to specifically repress IL-6. Nature. (2015) 525:389–93. doi: 10.1038/nature15252, PMID: 26287468 PMC4697747

[67] López-Moyado IF Ko M Hogan PG Rao A . TET enzymes in the immune system: from DNA demethylation to immunotherapy inflammation and cancer. Annu Rev Immunol. (2024) 42:455–88. doi: 10.1146/annurev-immunol-080223-044610, PMID: 38360546

[68] Chen L-L Morcelle C Cheng Z-L Chen X Xu Y Gao Y . Itaconate inhibits TET DNA dioxygenases to dampen inflammatory responses. Nat Cell Biol. (2022) 24:353–63. doi: 10.1038/s41556-022-00853-8, PMID: 35256775 PMC9305987

[69] Bambouskova M Potuckova L Paulenda T Kerndl M Mogilenko DA Lizotte K . Itaconate confers tolerance to late NLRP3 inflammasome activation. Cell Rep. (2021) 34:108756. doi: 10.1016/j.celrep.2021.108756, PMID: 33691097 PMC8039864

[70] Liu H Feng Y Xu M Yang J Wang Z Di G . Four-octyl itaconate activates keap1-nrf2 signaling to protect neuronal cells from hydrogen peroxide. Cell Communication Signaling. (2018) 16:81. doi: 10.1186/s12964-018-0294-2, PMID: 30442144 PMC6238317

[71] Tada M Kudo Y Kono M Kanda M Takeyama S Sakiyama K . Itaconate reduces proliferation and migration of fibroblast-like synoviocytes and ameliorates arthritis models. Clin Immunol. (2024) 264:110255. doi: 10.1016/j.clim.2024.110255, PMID: 38763433

[72] Yan Z Martin SH Gotzek D Arsenault SV Duchen P Helleu Q . Evolution of a supergene that regulates a trans-species social polymorphism. Nat Ecol Evol. (2020) 4:240–9. doi: 10.1038/s41559-019-1081-1, PMID: 31959939

[73] Herbrich S Chaib M Anandhan S Andrewes SW Nagarajan A Guan B . TET2-mutant clonal hematopoiesis enhances macrophage antigen presentation and improves immune checkpoint therapy in solid tumors. Cancer Cell. (2025) 44 (1):187–202.e7. doi: 10.1016/j.ccell.2025.09.011, PMID: 41106379 PMC13746248

[74] De La Calle-Fabregat C Calafell-Segura J Gardet M Dunsmore G Mulder K Ciudad L . NF-κB and TET2 promote macrophage reprogramming in hypoxia that overrides the immunosuppressive effects of the tumor microenvironment. Sci Adv. (2024) 10:eadq5226. doi: 10.1126/sciadv.adq5226, PMID: 39292770 PMC11409945

[75] Jiang S . Tet2 at the interface between cancer and immunity. Commun Biol. (2020) 3:667. doi: 10.1038/s42003-020-01391-5, PMID: 33184433 PMC7661537

[76] Zhang X Qian S Wu P Yu B Yin D Peng X . Tumor-associated macrophage-derived itaconic acid contributes to nasopharyngeal carcinoma progression by promoting immune escape via TET2. Cell Commun Signal. (2024) 22:413. doi: 10.1186/s12964-024-01799-0, PMID: 39192276 PMC11348665

[77] Qin W Qin K Zhang Y Jia W Chen Y Cheng B . S-glycosylation-based cysteine profiling reveals regulation of glycolysis by itaconate. Nat Chem Biol. (2019) 15:983–91. doi: 10.1038/s41589-019-0323-5, PMID: 31332308

[78] Diotallevi M Ayaz F Nicol T Crabtree MJ . Itaconate as an inflammatory mediator and therapeutic target in cardiovascular medicine. Biochem Soc Trans. (2021) 49:2189–98. doi: 10.1042/BST20210269, PMID: 34665229 PMC8589439

[79] Priya M Gupta SK Koundal A Kapoor S Tiwari S Kidwai S . Itaconate mechanism of action and dissimilation in mycobacterium tuberculosis. Proc Natl Acad Sci USA. (2025) 122:e2423114122. doi: 10.1073/pnas.2423114122, PMID: 39841148 PMC11789021

[80] Naujoks J Tabeling C Dill BD Hoffmann C Brown AS Kunze M . IFNs modify the proteome of legionella-containing vacuoles and restrict infection via IRG1-derived itaconic acid. PloS Pathog. (2016) 12:e1005408. doi: 10.1371/journal.ppat.1005408, PMID: 26829557 PMC4734697

[81] Zhang C Huang T Li L . Targeting cuproptosis for cancer therapy: mechanistic insights and clinical perspectives. J Hematol Oncol. (2024) 17:68. doi: 10.1186/s13045-024-01589-8, PMID: 39152464 PMC11328505

[82] Yang W Wang Y Huang Y Yu J Wang T Li C . 4-octyl itaconate inhibits aerobic glycolysis by targeting GAPDH to promote cuproptosis in colorectal cancer. Biomed Pharmacother. (2023) 159:114301. doi: 10.1016/j.biopha.2023.114301, PMID: 36706634

[83] Luo Y Jiang L-Y Liao Z-Z Wang Y-Y Wang Y-D Xiao X-H . Metabolic regulation of inflammation: exploring the potential benefits of itaconate in autoimmune disorders. Immunology. (2025) 174:189–202. doi: 10.1111/imm.13875, PMID: 39542834

[84] Lang R Siddique MNAA . Control of immune cell signaling by the immuno-metabolite itaconate. Front Immunol. (2024) 15:1352165. doi: 10.3389/fimmu.2024.1352165, PMID: 38487538 PMC10938597

[85] Day EA O'Neill LAJ . Protein targeting by the itaconate family in immunity and inflammation. Biochem J. (2022) 479:2499–510. doi: 10.1042/BCJ20220364, PMID: 36546613

[86] He R Liu B Xiong R Geng B Meng H Lin W . Itaconate Inhibits Ferroptosis of Macrophage via Nrf2 Pathways against Sepsis-Induced Acute Lung Injury. Cell Death Discov. (2022) 8:43. doi: 10.1038/s41420-021-00807-3, PMID: 35110526 PMC8810876

[87] Zhang P Wang Y Yang W Yin Y Li C Ma X . 4-octyl itaconate regulates immune balance by activating nrf2 and negatively regulating PD-L1 in a mouse model of sepsis. Int J Biol Sci. (2022) 18:6189–209. doi: 10.7150/ijbs.74456, PMID: 36439878 PMC9682535

[88] Chen M Su W Chen F Lai T Liu Y Yu D . Mechanisms underlying the therapeutic effects of 4-octyl itaconate in treating sepsis based on network pharmacology and molecular docking. Front Genet. (2022) 13:1056405. doi: 10.3389/fgene.2022.1056405, PMID: 36406124 PMC9671214

[89] Pan J Zhao X Lin C Xu H Yin Z Liu T . Immune responsive gene 1 a novel oncogene increases the growth and tumorigenicity of glioma. Oncol Rep. (2014) 32:1957–66. doi: 10.3892/or.2014.3474, PMID: 25216059

[90] Li Y Zhang P Wang C Han C Meng J Liu X . Immune responsive gene 1 (IRG1) promotes endotoxin tolerance by increasing A20 expression in macrophages through reactive oxygen species. J Biol Chem. (2013) 288:16225–34. doi: 10.1074/jbc.M113.454538, PMID: 23609450 PMC3675562

[91] Konishi T Lentsch AB . Hepatic ischemia/reperfusion: mechanisms of tissue injury repair and regeneration. Gene Expr. (2017) 17:277–87. doi: 10.3727/105221617X15042750874156, PMID: 28893351 PMC5885149

[92] Luo S Luo R Deng G Huang F Lei Z . Programmed cell death from liver ischemia–reperfusion injury perspective: an overview. Heliyon. (2024) 10:e32480. doi: 10.1016/j.heliyon.2024.e32480, PMID: 39040334 PMC11260932

[93] Yi Z Deng M Scott MJ Fu G Loughran PA Lei Z . Immune-responsive gene 1/itaconate activates nuclear factor erythroid 2–related factor 2 in hepatocytes to protect against liver ischemia–reperfusion injury. Hepatology. (2020) 72:1394–411. doi: 10.1002/hep.31147, PMID: 31997373 PMC7702080

[94] Chouchani ET Pell VR Gaude E Aksentijević D Sundier SY Robb EL . Ischaemic accumulation of succinate controls reperfusion injury through mitochondrial ROS. Nature. (2014) 515:431–5. doi: 10.1038/nature13909, PMID: 25383517 PMC4255242

[95] Kula-Alwar D Prag HA Krieg T . Targeting succinate metabolism in ischemia/reperfusion injury. Circulation. (2019) 140:1968–70. doi: 10.1161/CIRCULATIONAHA.119.042791, PMID: 31815541

[96] Zhu D Zhao Y Luo Y Qian X Zhang Z Jiang G . Irg1-Itaconate Axis Protects against Acute Kidney Injury via Activation of Nrf2. Am J Trans Res. (2021) 13:1155–69., PMID: 33841646 PMC8014393

[97] Chen S Yao H Lou Y Wang H Xie B Wu J . Pharmacological upregulation of macrophage-derived itaconic acid by pubescenoside C attenuated myocardial ischemia–reperfusion injury. J Advanced Res. (2025) 74:571–87. doi: 10.1016/j.jare.2024.09.024, PMID: 39357647 PMC12302717

[98] Ma K Zhou P Zhang W Zeng L Tao K Zhang P . Itaconic acid: A regulator of immune responses and inflammatory metabolism. CIMB. (2025) 47:534. doi: 10.3390/cimb47070534, PMID: 40729001 PMC12293325

[99] Laffusa A Burti C Viganò C Poggi F Grieco L Occhipinti V . Inflammatory bowel disease: understanding therapeutic effects of distinct molecular inhibitors as the key to current and future advanced therapeutic strategies. Biomedicines. (2025) 13:2667. doi: 10.3390/biomedicines13112667, PMID: 41301759 PMC12649937

[100] Zhen Y Zhang H . NLRP3 inflammasome and inflammatory bowel disease. Front Immunol. (2019) 10:276. doi: 10.3389/fimmu.2019.00276, PMID: 30873162 PMC6403142

[101] Li X Li Q Xiong B Chen H Wang X Zhang D . Discoidin domain receptor 1(DDR1) promote intestinal barrier disruption in ulcerative colitis through tight junction proteins degradation and epithelium apoptosis. Pharmacol Res. (2022) 183:106368. doi: 10.1016/j.phrs.2022.106368, PMID: 35905891

[102] Rogler G Brand K Vogl D Page S Hofmeister R Andus T . Nuclear factor κB is activated in macrophages and epithelial cells of inflamed intestinal mucosa. Gastroenterology. (1998) 115:357–69. doi: 10.1016/S0016-5085(98)70202-1, PMID: 9679041

[103] Kim HW Yu A-R Lee JW Yoon HS Lee BS Park H-W . Aconitate decarboxylase 1 deficiency exacerbates mouse colitis induced by dextran sodium sulfate. IJMS. (2022) 23:4392. doi: 10.3390/ijms23084392, PMID: 35457208 PMC9025264

[104] Muro P Zhang L Li S Zhao Z Jin T Mao F . The emerging role of oxidative stress in inflammatory bowel disease. Front Endocrinol. (2024) 15:1390351. doi: 10.3389/fendo.2024.1390351, PMID: 39076514 PMC11284038

[105] Wang Y Zhao X Gao Y Zhao C Li J Wang S . 4-octyl itaconate alleviates dextran sulfate sodium-induced ulcerative colitis in mice via activating the KEAP1-NRF2 pathway. Inflammopharmacology. (2024) 32:2555–74. doi: 10.1007/s10787-024-01490-3, PMID: 38767761

[106] Li W Chen D Zhu Y Ye Q Hua Y Jiang P . Alleviating pyroptosis of intestinal epithelial cells to restore mucosal integrity in ulcerative colitis by targeting delivery of 4-octyl-itaconate. ACS Nano. (2024) 18:16658–73. doi: 10.1021/acsnano.4c01520, PMID: 38907726

[107] McNamara KM Latour YL Hawkins CV Williams KJ Barry DP Allaman MM . Aconitate decarboxylase 1 downregulates colitis and maintains homeostasis of the gut metabolome and microbiome. Gastro Hep Adv. (2025) 4:100748. doi: 10.1016/j.gastha.2025.100748, PMID: 41368208 PMC12683919

[108] Kachler K Andreev D Thapa S Royzman D Gießl A Karuppusamy S . Acod1-mediated inhibition of aerobic glycolysis suppresses osteoclast differentiation and attenuates bone erosion in arthritis. Ann Rheumatic Dis. (2024) 83:1691–706. doi: 10.1136/ard-2023-224774, PMID: 38964754 PMC11671873

[109] Braun T Zwerina J . Positive regulators of osteoclastogenesis and bone resorption in rheumatoid arthritis. Arthritis Res Ther. (2011) 13:235. doi: 10.1186/ar3380, PMID: 21861862 PMC3239343

[110] O’ Gradaigh D Ireland D Bord S Compston JE . Joint erosion in rheumatoid arthritis: interactions between tumour necrosis factor α Interleukin 1 and receptor activator of nuclear factor κB ligand (RANKL) regulate osteoclasts. Ann Rheumatic Dis. (2004) 63:354–9. doi: 10.1136/ard.2003.008458, PMID: 15020327 PMC1754946

[111] Schett G Gravallese E . Bone erosion in rheumatoid arthritis: mechanisms diagnosis and treatment. Nat Rev Rheumatol. (2012) 8:656–64. doi: 10.1038/nrrheum.2012.153, PMID: 23007741 PMC4096779

[112] Rong K Wang D Pu X Zhang C Zhang P Cao X . Inflammatory macrophage-derived itaconate inhibits DNA demethylase TET2 to prevent excessive osteoclast activation in rheumatoid arthritis. Bone Res. (2025) 13:60. doi: 10.1038/s41413-025-00437-w, PMID: 40500265 PMC12159140

[113] Sun X Zhang B Pan X Huang H Xie Z Ma Y . Octyl itaconate inhibits osteoclastogenesis by suppressing hrd1 and activating nrf2 signaling. FASEB J. (2019) 33:12929–40. doi: 10.1096/fj.201900887RR, PMID: 31490085 PMC6902740

[114] Xie Y Cheng Q Xu ML Xue J Wu H Du Y . Itaconate: A potential therapeutic strategy for autoimmune disease. Scandinavian J Immunol. (2025) 101:e70026. doi: 10.1111/sji.70026, PMID: 40289463

[115] Runtsch MC Angiari S Hooftman A Wadhwa R Zhang Y Zheng Y . Itaconate and itaconate derivatives target JAK1 to suppress alternative activation of macrophages. Cell Metab. (2022) 34:487–501.e8. doi: 10.1016/j.cmet.2022.02.002, PMID: 35235776

[116] Kieler M Prammer LS Heller G Hofmann M Sperger S Hanetseder D . Itaconate is a metabolic regulator of bone formation in homeostasis and arthritis. Ann Rheumatic Dis. (2024) 83:1465–79. doi: 10.1136/ard-2023-224898, PMID: 38986577 PMC11503170

[117] Li Z Zheng W Kong W Zeng T . Itaconate: A potent macrophage immunomodulator. Inflammation. (2023) 46:1177–91. doi: 10.1007/s10753-023-01819-0, PMID: 37142886 PMC10159227

[118] Xiu C Luo H Huang W Fan S Yuan C Chen J . Lobetyolin suppressed osteoclastogenesis and alleviated bone loss in ovariectomy-induced osteoporosis via hindering P50/P65 nuclear translocation and downstream NFATc1/c-fos expression. Drug Design, Development and Therapy (2025) 19:4689–715. doi: 10.2147/DDDT.S515930, PMID: 40486125 PMC12145116

[119] Manara M Sinigaglia L . Bone and TNF in rheumatoid arthritis: clinical implications. RMD Open. (2015) 1:e000065. doi: 10.1136/rmdopen-2015-000065, PMID: 26557382 PMC4632149

[120] Bargagli E Piccioli C Rosi E Torricelli E Turi L Piccioli E . Pirfenidone and nintedanib in idiopathic pulmonary fibrosis: real-life experience in an italian referral centre. Pulmonology. (2019) 25:149–53. doi: 10.1016/j.pulmoe.2018.06.003, PMID: 30236523

[121] Wynn T . Cellular and molecular mechanisms of fibrosis. J Pathol. (2008) 214:199–210. doi: 10.1002/path.2277, PMID: 18161745 PMC2693329

[122] Ogger PP Albers GJ Hewitt RJ O’Sullivan BJ Powell JE Calamita E . Itaconate controls the severity of pulmonary fibrosis. Sci Immunol. (2020) 5:eabc1884. doi: 10.1126/sciimmunol.abc1884, PMID: 33097591 PMC7116646

[123] Han Y-Y Gu X Yang C-Y Ji H-M Lan Y-J Bi Y-Q . Protective Effect of Dimethyl Itaconate against Fibroblast–Myofibroblast Differentiation during Pulmonary Fibrosis by Inhibiting TXNIP. J Cell Physiol. (2021) 236:7734–44. doi: 10.1002/jcp.30456, PMID: 34061990

[124] Wang Y Wei J Deng H Zheng L Yang H Lv X . The role of nrf2 in pulmonary fibrosis: molecular mechanisms and treatment approaches. Antioxidants. (2022) 11:1685. doi: 10.3390/antiox11091685, PMID: 36139759 PMC9495339

[125] Wu Y Zhang Y Jiang F He S Zhang Y Chen D . 4-OI ameliorates bleomycin-induced pulmonary fibrosis by activating nrf2 and suppressing macrophage-mediated epithelial-mesenchymal transition. Inflammation Res. (2023) 72:1133–45. doi: 10.1007/s00011-023-01733-z, PMID: 37169970

[126] He R Zuo Y Yi K Liu B Song C Li N . The role and therapeutic potential of itaconate in lung disease. Cell Mol Biol Lett. (2024) 29:129. doi: 10.1186/s11658-024-00642-1, PMID: 39354366 PMC11445945

[127] Weiss JM . The promise and peril of targeting cell metabolism for cancer therapy. Cancer Immunol Immunother. (2020) 69:255–61. doi: 10.1007/s00262-019-02432-7, PMID: 31781842 PMC7004869

[128] Chen F Dowerg B Cordes T . The yin and yang of itaconate metabolism and its impact on the tumor microenvironment. Curr Opin Biotechnol. (2023) 84:102996. doi: 10.1016/j.copbio.2023.102996, PMID: 37806082

[129] Weiss JM Davies LC Karwan M Ileva L Ozaki MK Cheng RYS . Itaconic acid mediates crosstalk between macrophage metabolism and peritoneal tumors. J Clin Invest. (2018) 128:3794–805. doi: 10.1172/JCI99169, PMID: 29920191 PMC6118601

[130] Zhao H Teng D Yang L Xu X Chen J Jiang T . Myeloid-derived itaconate suppresses cytotoxic CD8+ T cells and promotes tumour growth. Nat Metab. (2022) 4:1660–73. doi: 10.1038/s42255-022-00676-9, PMID: 36376563 PMC10593361

[131] Wang Q Li XL Mei Y Ye J-C Fan W Cheng G-H . The anti-inflammatory drug dimethyl itaconate protects against colitis-associated colorectal cancer. J Mol Med. (2020) 98:1457–66. doi: 10.1007/s00109-020-01963-2, PMID: 32840638

[132] Fan Y Dan W Wang Y Ma Z Jian Y Liu T . Itaconate transporter SLC13A3 confers immunotherapy resistance via alkylation-mediated stabilization of PD-L1. Cell Metab. (2025) 37:514–526.e5. doi: 10.1016/j.cmet.2024.11.012, PMID: 39809284

[133] Lin H Tison K Du Y Kirchhoff P Kim C Wang W . Itaconate transporter SLC13A3 impairs tumor immunity via endowing ferroptosis resistance. Cancer Cell. (2024) 42:2032–2044.e6. doi: 10.1016/j.ccell.2024.10.010, PMID: 39515327 PMC11631639

[134] Gautam AK Kumar P Raj R Kumar D Bhattacharya B Rajinikanth PS . Preclinical Evaluation of Dimethyl Itaconate against Hepatocellular Carcinoma via Activation of the e/iNOS-Mediated NF-κB–Dependent Apoptotic Pathway. Front Pharmacol. (2022) 12:823285. doi: 10.3389/fphar.2021.823285, PMID: 35095533 PMC8795766

[135] Wang Z Cui L Lin Y Huo B Zhang H Xie C . Cancer cell-intrinsic biosynthesis of itaconate promotes tumor immunogenicity. EMBO J. (2024) 43:5530–47. doi: 10.1038/s44318-024-00217-y, PMID: 39349845 PMC11574104

[136] Schofield JH Longo J Sheldon RD Albano E Ellis AE Hawk MA . Acod1 expression in cancer cells promotes immune evasion through the generation of inhibitory peptides. Cell Rep. (2024) 43:113984. doi: 10.1016/j.celrep.2024.113984, PMID: 38520689 PMC11090053

[137] Wang C Wen J Yan Z Zhou Y Gong Z Luo Y . Suppressing neutrophil itaconate production attenuates mycoplasma pneumoniae pneumonia. PloS Pathog. (2024) 20:e1012614. doi: 10.1371/journal.ppat.1012614, PMID: 39499730 PMC11567624

[138] Kang H Liu T Wang Y Bai W Luo Y Wang J . Neutrophil–macrophage communication via extracellular vesicle transfer promotes itaconate accumulation and ameliorates cytokine storm syndrome. Cell Mol Immunol. (2024) 21:689–706. doi: 10.1038/s41423-024-01174-6, PMID: 38745069 PMC11637192

[139] Ramalho T Assis PA Ojelabi O Tan L Carvalho B Gardinassi L . Itaconate impairs immune control of plasmodium by enhancing mtDNA-mediated PD-L1 expression in monocyte-derived dendritic cells. Cell Metab. (2024) 36:484–497.e6. doi: 10.1016/j.cmet.2024.01.008, PMID: 38325373 PMC10940217

[140] Yang X Deng Y Ye Y Meng J Su M Wei W . Macrophage-derived itaconate suppresses dendritic cell function to promote acquired resistance to anti-PD-1 immunotherapy. Cancer Res. (2025) 85(10):1842–56. doi: 10.1158/0008-5472.CAN-24-2982, PMID: 40036156

[141] Wei W Yang X Chen Y Che M Ye Y Deng Y . Boosting mRNA Cancer Vaccine Efficacy via Targeting Irg1 on Macrophages in Lymph Nodes. Theranostics. (2025) 15:6329–46. doi: 10.7150/thno.110305, PMID: 40521193 PMC12159843

